# Six immune-related promising biomarkers may promote hepatocellular carcinoma prognosis: a bioinformatics analysis and experimental validation

**DOI:** 10.1186/s12935-023-02888-9

**Published:** 2023-03-23

**Authors:** Xia-Hui Lin, Dong-ping Li, Zhi-Yong Liu, Si Zhang, Wen-qing Tang, Rong-xin Chen, Shu-qiang Weng, Yu-jen Tseng, Ru-yi Xue, Ling Dong

**Affiliations:** 1 Department of Gastroenterology and Hepatology, Zhongshan Hospital, Fudan University, Shanghai, 200032 China; 2 Shanghai Institute of Liver Disease, Shanghai, 200032 China; 3 Key Laboratory of Glycoconjugate Research Ministry of Public Health, Department of Biochemistry and Molecular Biology, School of Basic Medical Sciences, Fudan University, Shanghai, 200032 China; 4 Key Laboratory of Carcinogenesis and Cancer Invasion, Liver Cancer Institute, Zhongshan Hospital, Fudan University, Shanghai, 200032 China; 5 Department of Digestive Diseases, Huashan Hospital, Fudan University, 12 Wulumuqi Middle Road, Shanghai, 200040 China

**Keywords:** Hepatocellular carcinoma, Prognosis, Immune gene signature, Competing endogenous RNA

## Abstract

**Background:**

Abnormal miRNA and mRNA expression and dysregulated immune microenvironment have been found to frequently induce the progression of hepatocellular carcinoma (HCC) in recent reports. In particular, the immune-related competing endogenous RNAs (ceRNA) mechanism plays a crucial role in HCC progression. However, the underlying mechanisms remain unclear.

**Methods:**

Differentially expressed immune-related genes were obtained from the Immport, GEO, and TCGA databases. The mRNA and protein expression levels in HCC tissues and adjacent normal tissues were confirmed, and we further investigated the methylation levels of these biomarkers to explore their function. Then, the TIMER and TISCH databases were used to assess the relationship between immune infiltration and hub genes. Survival analysis and univariate and multivariate Cox models were used to evaluate the association between hub genes and HCC diagnosis. Hub gene expression was experimentally validated in six HCC cell lines and 15 HCC samples using qRT-PCR and immunohistochemistry. The hub genes were uploaded to DSigDB for drug prediction enrichment analysis.

**Results:**

We identified that patients with abnormal miRNAs (hsa-miR-125b-5p and hsa-miR-21-5p) and their targeted genes (NTF3, PSMD14, CD320, and SORT1) had a worse prognosis. Methylation analysis of miRNA-targeted genes suggested that alteration of methylation levels is also a factor in the induction of tumorigenesis. We also found that the development of HCC progression caused by miRNA-mRNA interactions may be closely correlated with the infiltration of immunocytes. Moreover, the GSEA, GO, and KEGG analysis suggested that several common immune-related biological processes and pathways were related to miRNA-targeted genes. The results of qRT-PCR, immunohistochemistry, and western blotting were consistent with our bioinformatics results, suggesting that abnormal miRNAs and their targeted genes may affect HCC progression.

**Conclusions:**

Briefly, our study systematically describes the mechanisms of miRNA-mRNA interactions in HCC and predicts promising biomarkers that are associated with immune filtration for HCC progression.

**Supplementary Information:**

The online version contains supplementary material available at 10.1186/s12935-023-02888-9.

## Background

Cancer is not only the second leading cause of death in people after heart disease but also the first or second leading cause of death for every age group [1]. Hepatocellular carcinoma (HCC) is one of the most common cancers worldwide. Although surgical resection, liver transplantation, radiotherapy, and chemotherapy have improved the survival rate of patients with HCC, most patients are diagnosed with HCC at an advanced stage and are unsuitable for surgery due to the concealment of HCC and the lack of specific early biomarkers [2–4]. Therefore, there is an urgent need to identify novel prognostic biomarkers and/or therapeutic targets to bridge the gap in the diagnosis, prevention, and treatment of HCC [5].

As is well known, public data can provide us with a large amount of clinical data and genetic data that can help us deeply mine the biomarkers (miRNA, mRNA, etc.) of diseases and study their pathogenesis. It has been reported that miRNA-mRNA interactions can regulate the progression of many diseases, including HCC [6–8]. Previous studies have focused on this point and revealed that the ceRNA network is involved in HCC growth, metastasis, and prognosis; for example, Xu et al. found that miR-885-5p can regulate HCC progression by silencing hexokinase 2 [9]. Xiao et al. confirmed that miR-330-5p can promote HCC progression by targeting SPRY2 [10]. In addition, secreted miRNAs have been reported to act in a paracrine manner in the surrounding microenvironment to promote tumor progression [11]. In addition, the existence of tumor-infiltrating immune cells, such as macrophages [12] and lymphocytes [13], is evidence of immune system activity and is thought to play a crucial role in cancer growth, metastasis, and progression. However, studies focusing on the regulatory mechanisms between ceRNA networks and immune infiltration in HCC remain unclear.

**Fig. 1:**
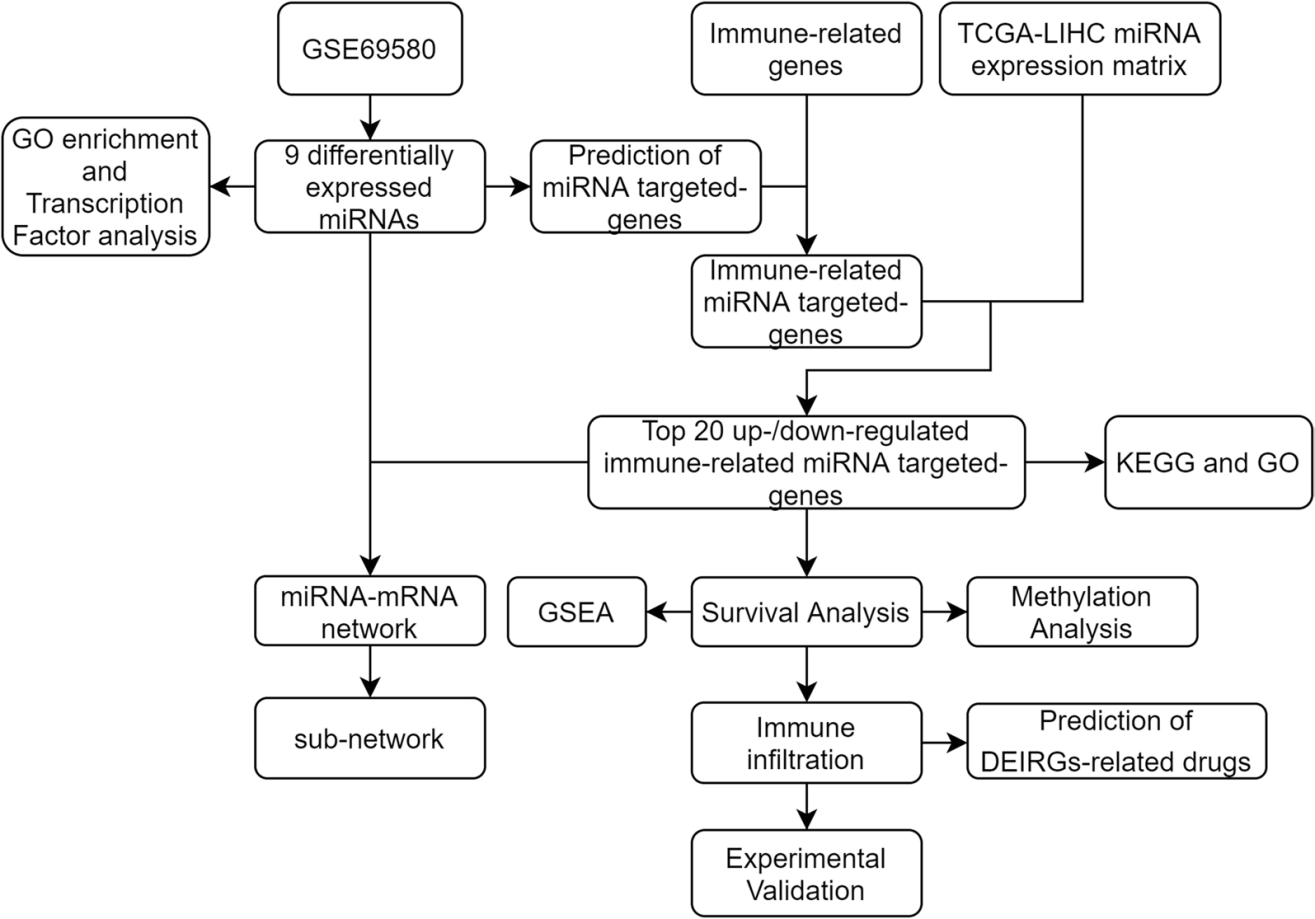
Flow chart of the whole study

In our study, we first identified differentially expressed miRNAs (DEMis) and immune-related genes (DEIRGs). KEGG and GO enrichment analyses were performed to investigate the functions of DEMis and DEIRGs. Survival analysis was performed to screen for key miRNA-targeted genes. The protein expression and methylation levels of hub genes were confirmed by immunohistochemistry using the HPA and ULCAN databases. The TIMER and Cell Marker databases were used to explore the correlation between hub gene signatures and immune cells. The signals of key genes were assessed using gene set enrichment analysis (GSEA). We then conducted univariate and multivariate Cox regression models to screen for novel prognostic markers and confirm the independent prognostic role of the hub gene signature. A nomogram was developed to predict the outcomes of HCC. Finally, we performed a qRT-PCR assay to confirm the expression of miRNAs, and western blot and immunohistochemistry assays to confirm the expression of miRNA-targeted genes. The flow chart of our study is presented in Fig. 1. In summary, our study screened several novel biomarkers that could be used as prognostic predictors of HCC.

## Materials and methods

### Data processing

The TCGA dataset (including 50 normal and 374 tumor patients) was collected from The Cancer Genome Atlas (TCGA) project (https://portal.gdc.cancer.gov/). Gene expression in fragments per kilobase per million (FPKM) format was converted to transcript per million reads (TPM).

Additionally, the GSE69580 array dataset (Platform: GPL10850) was accessed from the Gene Expression Omnibus (GEO) database (https://www.ncbi.nlm.nih.gov/geo/), including five tumors and five normal samples. Each gene was normalized to the median, and the data were quantile-normalized. GSE14520 and GSE76427 were used to validate the expression levels of the hub genes.

A list of immune-related gene was downloaded from the ImmPort portal database (https://www.immport.org/home).

### Differential analysis of miRNAs and mRNAs

Using the “limma” R package [14] to screen differentially expressed miRNAs (DEMis) and mRNAs (DEMs) with the thresholds of |logFC| > 0.5 and *p* adj < 0.05. The volcano plots and heatmap were drawn by the “ggplot2” R packages.

### Construction of the miRNA-mRNA network

Based on the mechanism by which miRNAs can directly bind mRNAs to suppress the translation process in the cytoplasm, the steps for constructing an miRNA-mRNA network are as follows: (1) miRecords (https://www.hsls.pitt.edu/obrc/index.php?page=URL1237998207), miRTarbase (http://mirtarbase.cuhk.edu.cn/php/index.php), and Tarbase (http://carolina.imis.athenainnovation.gr/diana_tools/web/index.php?Ther=tarbasev8%2Findex) database was used to retrieve the validated miRNA-mRNA interaction pairs; (2) The Venny 2.1.0 online website (https://bioinfogp.cnb.csic.es/tools/venny/) was used to obtain the overlapping genes between the miRNAs’ validated targets, immune-related genes, and TCGA DEMs; (3) the overlapping genes were imported into the String (https://www.string-db.org/) database for the construction of the PPI network [15]; and (4) the miRNA–mRNA network was integrated.

### Functional enrichment analysis

To further explore the biological mechanisms of the promising biomarkers, we conducted Kyoto Encyclopedia of Genes and Genomes (KEGG) and Gene Ontology (GO) analysis. The FunRich software (version 3.1.3) was used to perform functional enrichment analysis of DEMis [16]. Functional enrichment analysis of DEMs was performed using the “ClusterProfiler” R package [17]. The bubble plots and Gocircle plots were visualized by the “ggplot2” R package. GSEA analysis was performed using the GSEA software (version 4.1.0) [18].

### Methylation analysis

DNA methylation is a critical epigenetic process that controls gene expression and behavior of cancer cells. UALCAN (http://ualcan.path.uab.edu/) was used to assess the methylation levels of hub genes in HCC and normal tissues, and MEXPRESS (http://mexpress.be) was used to evaluate the relationship between gene expression and DNA methylation.

### Immunohistochemistry and immune infiltrate analysis

The Human Protein Atlas (https://www.proteinatlas.org/) was used to assess the protein expression levels in HCC and normal tissues. The correlation between gene expression and tumor-infiltrating immune cells (B cells, CD8+ T cells, CD4+ T cells, macrophages, neutrophils, and dendritic cells) was evaluated using the Tumor Immune Estimation Resource (TIMER) database (https://cistrome.shinyapps.io/timer/) and tumor immune single-cell hub (TISCH) database (http://tisch.comp-genomics.org/home/).

Slides were dewaxed and rehydrated in a gradient of xylene and ethanol, then treated with citric acid epitope repair reagent at 100 °C for 20 min and cooled to room temperature to inhibit endogenous peroxidase activity. The cells were incubated with 5% bovine serum albumin (BSA) at room temperature for 30 min. Subsequently, the cells were incubated with the primary antibody (CD320: Santa Cruz sc-393892 Mouse 1:100; PSMD14: ABclonal A9608 Rabbit 1:100; SORT1: Santa Cruz sc-376561 Mouse 1:100; NTF3: ABclonal A12476 Rabbit 1:100); overnight at 4 °C. The next day, the cells were incubated with the corresponding HRP-labeled secondary antibody for 1 h. Finally, the sections were stained with diaminobenzidine (DAB) and observed under a microscope.

### Survival analysis and prognostic model

The survival curves of hub genes were obtained from the GEPIA database (http://gepia.cancer-pku.cn/index.html). The prognosis model (univariate and multivariate Cox regression analysis) was analyzed using the “Survival” and “Survminer” R packages. A nomogram has been used to predict cancer prognosis [19]. The “ggplot2” and “RMS” R packages were used to visualize the analysis results of the prognosis model. Statistical significance was set at *p* < 0.05.

### Cell lines and cell culture

Human liver cells L0-2, HCC cells (PLC/PRF/5, HepG2, and Hep3B) (Cell Bank of the Chinese Academy of Sciences, Shanghai, China), MHCC97H and HCCLM3 (Liver Cancer Institute, Fudan University, Shanghai, China), and Huh7 (Japanese Cancer Research Resources Bank) were cultured in Dulbecco’s modified Eagle’s medium (Gibco) supplemented with 10% fetal bovine serum (Gibco) and 1% penicillin–streptomycin (Invitrogen). Cell cultures were performed in a thermostatic incubator at 37 °C in a humidified atmosphere of 95% air and 5% CO_2_.

### Human samples

Ethical approval was obtained from the Zhongshan Hospital of Fudan University (Shanghai, China), and written informed consent was obtained from each patient. HCC and matched non-tumor liver tissues were collected from 15 patients who underwent curative resection at the Liver Cancer Institute, Zhongshan Hospital of Fudan University (Shanghai, China) in 2015. A pathological diagnosis of HCC was confirmed. Clinicopathological information was retrieved from the medical records.

### Quantitative reverse-transcription polymerase chain reaction (qRT-PCR) assay

Total RNA, including miRNA, was extracted from cells or tissues using TRIzol Reagent (Invitrogen), and cDNA was synthesized from RNA using the Reverse Transcription Kit (Takara). Subsequently, cDNA was amplified using the Maxinma SYBR Green qPCR Master Mix (Thermo Scientific). Target genes were quantified using the 2^−ΔΔCt^ method with glyceraldehyde-3-phosphate dehydrogenase (GAPDH) for normalization. Melting curve analysis was performed to assess the specificity of PCR products. The NTF3, SORT1, CD320, and PSMD14 primers were used for real-time PCR. Primers for qRT-PCR were purchased from Genepharma (Shanghai, China) and the sequences were as follows: CD320, forward: 5′-CGATGAGGAGGAGTGCAGGATT-3′, reverse: 5′-CATGGTTGTGGCATTCCTGAG-3′; PSMD14, forward: 5′-GAAGCCTTGTCGGAGAGAGC-3′, reverse: 5′-TGCCTGGATAGATGGCTTGT-3′; SORT1, forward: 5′-TCTCAGAGCCGAATGCCGTAGG-3′, reverse: 5′-GGTCCTTCCAGCATCTTTGTCCAG-3′; NTF3, forward: 5′-TGGTTACTTTTGCCACGATCT-3′, reverse: 5′-GGTGTCCATTGCAATCACCG-3′. The levels of miRNAs were measured by qRT-PCR using miDETECT A Track™ miRNA qRT-PCR Kit (RiboBio, Guangzhou, China) and performed on an ABI 7500 System (Applied Biosystems). The primers for hsa-miR-125b-5p, hsa-miR-21-5p, and U6 small nuclear RNA were obtained from RiboBio Company (Guangzhou, China). The sequences were covered by patents. miRNA expression was normalized to the expression of internal control U6 using the 2^−ΔΔCT^ method.

### Western blot

Proteins were extracted from cells or tissues using RIPA cell lysis with Protease Inhibitor Cocktail (Beyotime Biotechnology). The proteins were quantified using the BCA kit, subjected to 10% SDS-PAGE for separation, and transferred to 0.45 μM PVDF membranes (Millipore, USA). The membrane was incubated with the corresponding primary antibodies (CD320: Santa Cruz sc-393892 Mouse 1:500; PSMD14: ABclonal A9608 Rabbit 1:1000; SORT1: Santa Cruz sc-376561 Mouse 1:500; NTF3: ABclonal A12476 Rabbit 1:1000; GAPDH: Servicebio GB11002 Rabbit 1:1000; mTOR: CST #2983S Rabbit 1:1000; p-mTOR: CST #5536 Rabbit 1:1000; LC3B: CST #2775S Rabbit 1:1000; p62: CST #23214 Rabbit 1:1000; ATG5: CST #12994S Rabbit 1:1000) at 4 °C overnight, after blocking with 5% skim milk. followed by incubation with the corresponding HRP-conjugated secondary antibody (PeproTech), and the bands were visualized by enhanced chemiluminescence. The intensity of protein expression was measured using ImageJ software.

### Transwell migration and invasion assay

For invasion assay, 1 × 10^5^ cells suspended in serum-free medium were seeded into the upper chamber coated with 1 µg/µl Matrigel (BD Biosciences, USA) in 24-well transwell plates (8-μm pore size, Corning, NY, USA), and 600 μl DMEM with 10% FBS was added into the lower chamber. After incubation for an indicated time points at 37 °C in 5% CO_2_, the migrating and invading cells on the outer side of the upper chamber membrane were then fixed with 4% paraformaldehyde, stained with crystal violet and counted under a light microscope (100× magnification) in eight randomly selected areas.

### Statistical analysis

R software (version 4.0.3), GraphPad Prism (version 6.0), and SPSS (version 21.0) were used for the statistical analysis of the experimental data. Continuous data were compared using the Student’s t-test. Categorical data between the groups were compared using the chi-square test. The survival values of DEIRGs were evaluated using Kaplan–Meier (K–M) analysis. Statistical significance was set at *p* < 0.05.

## Results

### Identification of differentially expressed miRNAs in HCC

**Fig. 2:**
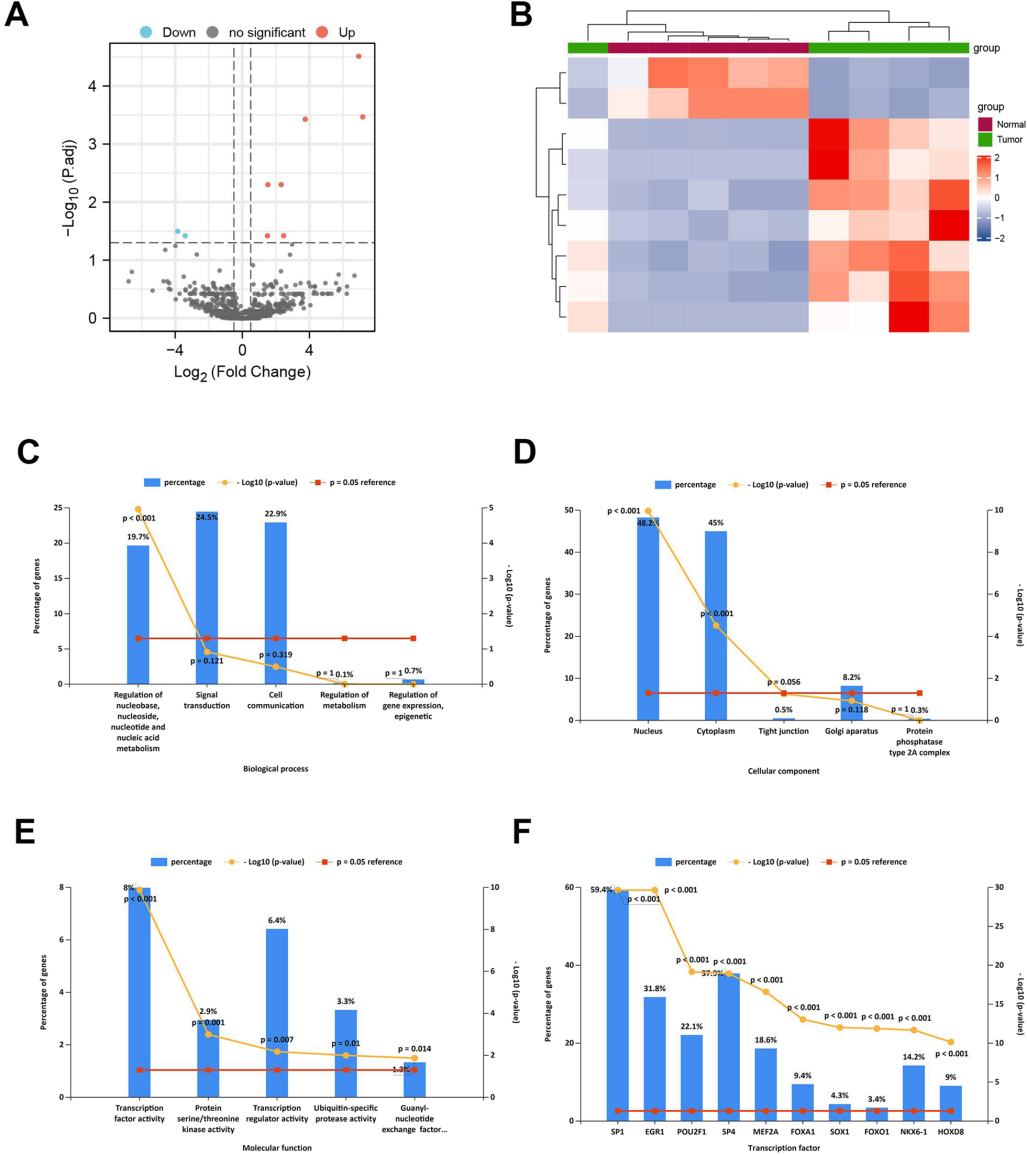
Identification of differentially expressed miRNAs in HCC. **A** The volcano plot shows differentially expressed genes (DEGs) (|log2FC| > 0.5, adj P-value < 0.05). **B** The heatmap shows the different expression level of DEGs. **C** The biological process terms of miRNAs. **D** The cellular component terms of miRNAs. **E** The molecular function terms of miRNAs. **F** The transcription factors enrichment analysis of miRNAs

The miRNA microarray data (GSE69580) containing five HCC tumors and five normal tissues were first quantile-normalized before data analysis (Additional file 1: Fig. S1). The filtering criteria were as described previously (|log2FC| > 0.5, adjusted *p* < 0.05). We identified seven upregulated and two downregulated miRNAs (Table 1). Volcano and heatmaps were drawn to show the differential expression of the nine miRNAs between the tumor and normal tissues (Fig. 2A, B). We then imported nine miRNAs into FunRich (3.1.3) software to perform miRNA GO enrichment analysis. The GO biological process terms (BP) showed that most miRNAs were involved in the regulation of nucleobase, nucleoside, nucleotide, and nucleic acid metabolism (19.7%, *p* < 0.001) (Fig. 2C). Regarding the GO cellular component terms, most miRNAs may be localized in the nucleus (48.2%, *p* < 0.001) and cytoplasm (45%, P < 0.001) (Fig. 2D). The GO molecular function terms showed that most of the miRNAs were associated with transcription factor activity (8%, *p* < 0.001), protein serine/threonine kinase (2.9%, P = 0.001), transcription regulator activity (6.4%, P = 0.007), ubiquitin-specific protease activity (3.3%, P = 0.01), and guanyl-nucleotide exchange factor (1.3%, P = 0.014) (Fig. 2E). For transcription factor analysis, we found that most miRNAs were related to transcription factors (TFs). We chose the top 10 TFs that were closely related to miRNAs. As shown in Fig. 2F, the TFs SP1, EGR1, POU2F1, SP4, MEF2A, FOXA1, SOX1, FOXO1, NKX6-1, and HOXD8 are associated with differentially expressed miRNAs.

**Table 1:** Differentially expressed miRNAs in miRNA microarray data (GSE69580)

miRNA	log2FC	P value	adj P value
hsa-miR-501-3p	6.942884019	3.29E−08	3.08E−05
hsa-miR-652	7.179979898	7.25E−07	0.000339885
hsa-miR-21	3.751696865	1.20E−06	0.00037339
hsa-miR-25	1.526300306	2.45E−05	0.005008273
hsa-miR-106b	2.31647731	2.67E−05	0.005008273
hsa-miR-19b	2.464975508	0.000321295	0.037983968
hsa-miR-93	1.500181836	0.000364841	0.037983968
hsa-miR-125b	− 3.854404135	0.000204187	0.031887224
hsa-miR-199a-5p	− 3.409947962	0.000341212	0.037983968

### Identification of differentially expressed immune-related miRNA targeted-genes

**Fig. 3:**
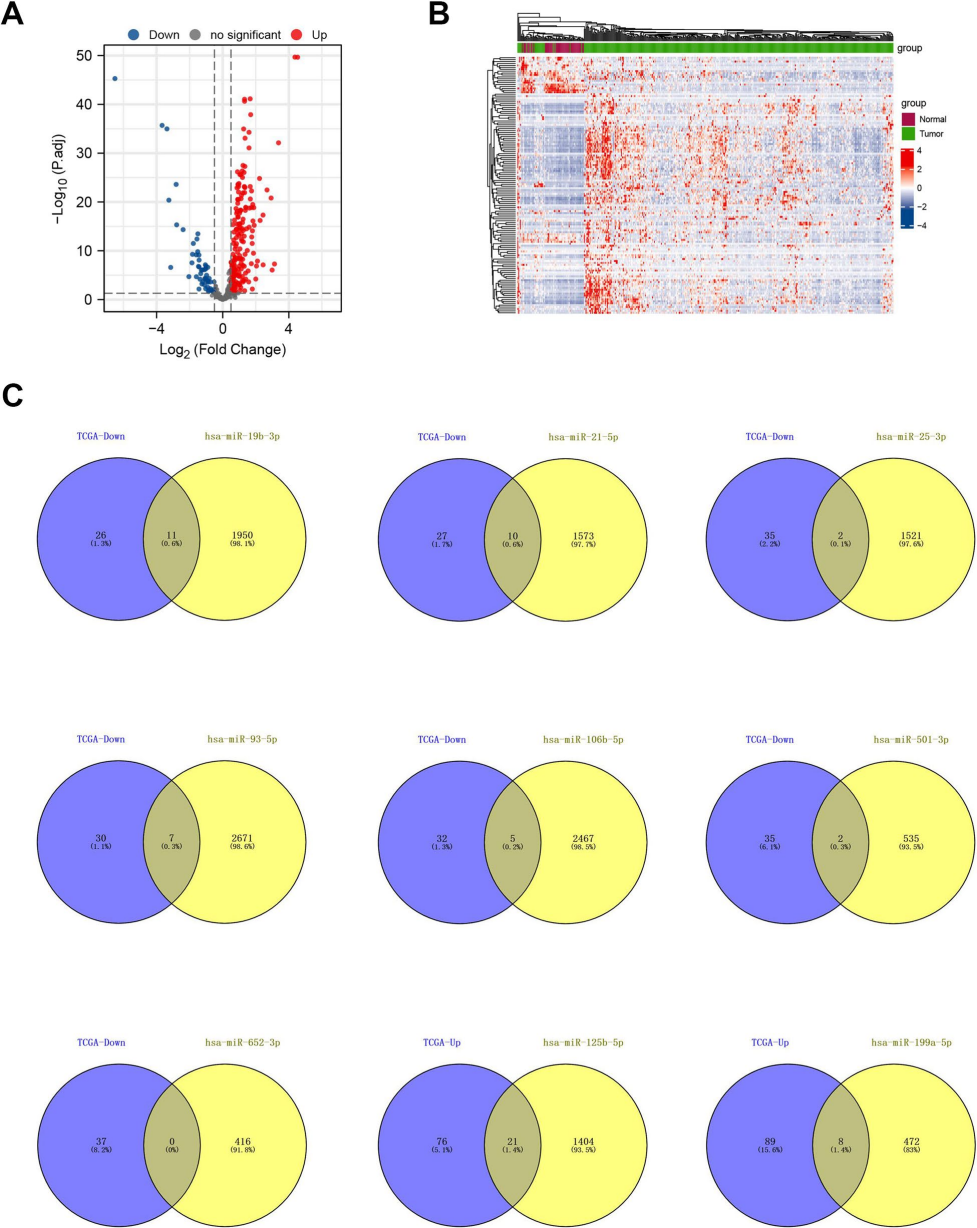
Identification of differentially expressed immune-related miRNA targeted-genes. **A** The volcano plot shows the differentially expressed mRNAs (DGMis). **B** The heatmap shows the different expression level of DEMis. **C** The Venn diagrams show the overlapping genes between the up-/down-regulated mRNAs in HCC tumor tissues and the targets of down-/up-regulated miRNA

We first uploaded nine miRNAs to three databases (miRecords, miRTarbase, and Tarbase), and then aggregated all validated interacting target genes in these three databases. After that, the final 393 immune-related target mRNA genes were obtained by screening overlapping genes between miRNA targeted genes and the list of genes downloaded from the ImmPort Portal database (Additional file 1: Fig. S2). We then extracted the expression matrix of these 393 genes from the TCGA database and normalized it before differential gene analysis (Additional file 1: Fig. S3). The filter criteria are |log2FC| > 1 and adjusted p-value < 0.05. We confirmed that 97 genes were upregulated and 37 genes were downregulated in HCC tumor tissues. Volcano and heatmaps were drawn to show the differential expression of these genes (Fig. 3A, B). Ultimately, we obtained the final genes by screening the overlapping genes between the upregulated/downregulated mRNAs in HCC tumor tissues and the targets of downregulated/upregulated miRNAs (Fig. 3C).

### Establishment of the network of miRNA-mRNA interaction

**Fig. 4:**
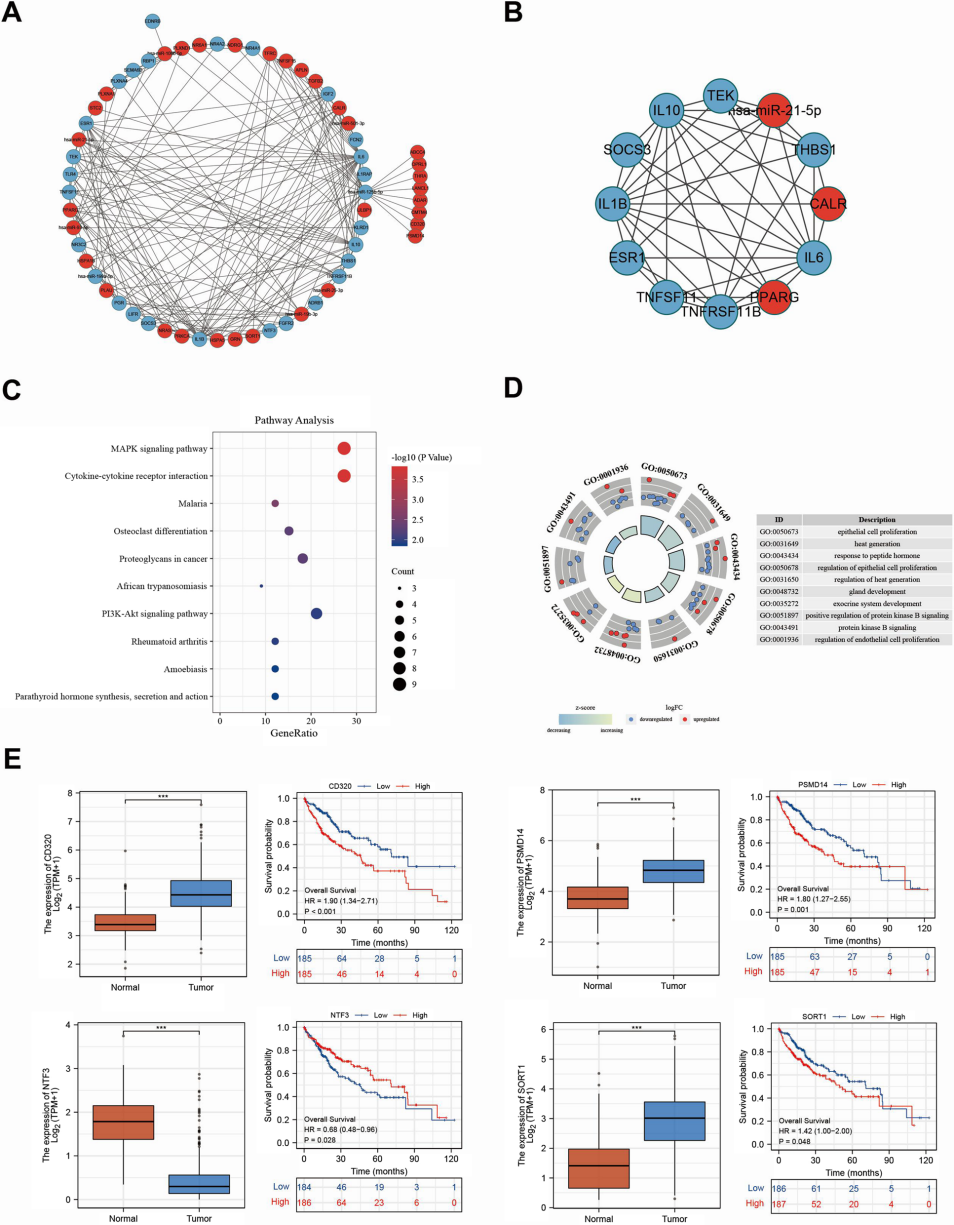
Establishment of the network of miRNA-mRNA interaction. **A** Network diagram composed of miRNA and top 20 up-/down-regulated. **B** The sub-network of miRNA-mRNA network. **C**, **D** The KEGG and GO analysis of top 20 up-/down-regulated mRNAs. **E** The overall survival of DEMs. (*P < 0.05, **P < 0.01, ***P < 0.001, ****P < 0.0001, n.s. not statistically significant)

The top 20 upregulated and downregulated mRNAs were chosen from the overlapping gene list for further analysis (Tables 2 and 3). As shown in Fig. 4A, B, we constructed the miRNA-mRNA network and utilized the MCODE plugin of Cytoscape software to obtain the sub-network from the whole network. Next, KEGG and GO analyses were performed using the “ClusterProfiler” R package. The results showed that the majority of the genes were enriched in the MAPK signaling pathway, cytokine-cytokine receptor interaction, and PI3K-Akt signaling pathway, etc. (Fig. 4C). Furthermore, most of the genes were related to GO:0050673 (epithelial cell proliferation), GO:0031649 (heat generation), and GO:0043434 (response to peptide hormone) (Fig. 4D). Subsequently, survival analyses of 40 genes were performed. Genes with a p-value < 0.05, were included in further analyses. Therefore, 15 miRNA targets were identified. We then retrieved studies and papers about the 15 miRNA targets in PubMed, and ultimately obtained four targets (NTF3, PSMD14, SORT1, and CD320) that had not been previously studied in-depth in HCC, especially the relationship between immune infiltration and HCC progression (Fig. 4E, Additional file 1: Fig. S4). Finally, we established a ceRNA network of hsa-miR-125b-5p-PSMD14/CD320/SORT1 and hsa-miR-21-5p-NTF3.

**Table 2:** The top 20 up-regulated mRNAs in 97 immune-related target upregulated genes

mRNA	log2FC	P value	Adj P value
APLN	4.099198436	8.06E−48	1.56E−45
STC2	3.438263368	1.85E−39	1.43E−37
ULBP1	2.038451905	9.84E−12	4.42E−11
TNFSF15	1.813937955	1.90E−15	1.22E−14
SORT1	1.702385929	2.80E−22	5.15E−21
PLXNA1	1.469397288	7.21E−27	2.53E−25
CMTM4	1.427441247	4.52E−19	4.84E−18
NR6A1	1.374408406	3.17E−14	1.75E−13
ABCC4	1.203128419	6.43E−07	1.62E−06
PLAU	1.18222759	2.19E−18	2.28E−17
TFRC	1.113029395	3.28E−16	2.48E−15
OPRL1	1.040268995	4.08E−10	1.51E−09
TGFB2	1.01873334	9.97E−06	2.20E−05
PRKCA	0.905992335	5.53E−13	2.81E−12
PLXND1	0.903769326	5.93E−16	4.01E−15
NDRG1	0.836579573	3.88E−06	8.97E−06
KL	0.825403262	0.000733615	0.00128716
CD320	0.774862617	3.39E−09	1.13E−08
THRA	0.731531843	7.49E−12	3.40E−11
PSMD14	0.715514554	6.06E−17	5.19E−16

**Table 3:** The top 20 down-regulated mRNAs in 37 immune-related target downregulated genes

mRNA	log2FC	P value	Adj P value
FCN2	− 7.268236784	2.86E−38	1.84E−36
NTF3	− 4.167516609	1.63E−27	6.28E−26
IGF2	− 3.792040278	3.02E−11	1.28E−10
ESR1	− 3.446868963	6.23E−26	1.85E−24
LIFR	− 3.150230613	3.48E−47	4.48E−45
TNFRSF17	− 2.233835877	0.001542129	0.002599396
NR4A1	− 2.173312708	1.67E−22	3.22E−21
SOCS3	− 2.070701175	8.99E−20	9.91E−19
TNFSF11	− 2.039241178	3.66E−08	1.04E−07
IL1RAP	− 2.003326002	8.96E−29	3.84E−27
TNFRSF11B	− 1.991456369	4.37E−10	1.61E−09
THBS1	− 1.858174887	5.66E−16	3.97E−15
NR4A2	− 1.810446793	1.76E−16	1.41E−15
IL1B	− 1.789334147	1.25E−13	6.71E−13
PLXNA4	− 1.740009766	7.69E−05	0.000149968
IL10	− 1.720330149	8.74E−09	2.70E−08
FGFR2	− 1.712538611	5.50E−05	0.000109468
ADRB1	− 1.705463538	1.61E−05	3.39E−05
TEK	− 1.58302732	3.44E−15	2.14E−14
EDNRB	− 1.514996456	1.75E−20	2.05E−19

### Correlation between miRNA targeted-genes expression and immune infiltration

**Fig. 5:**
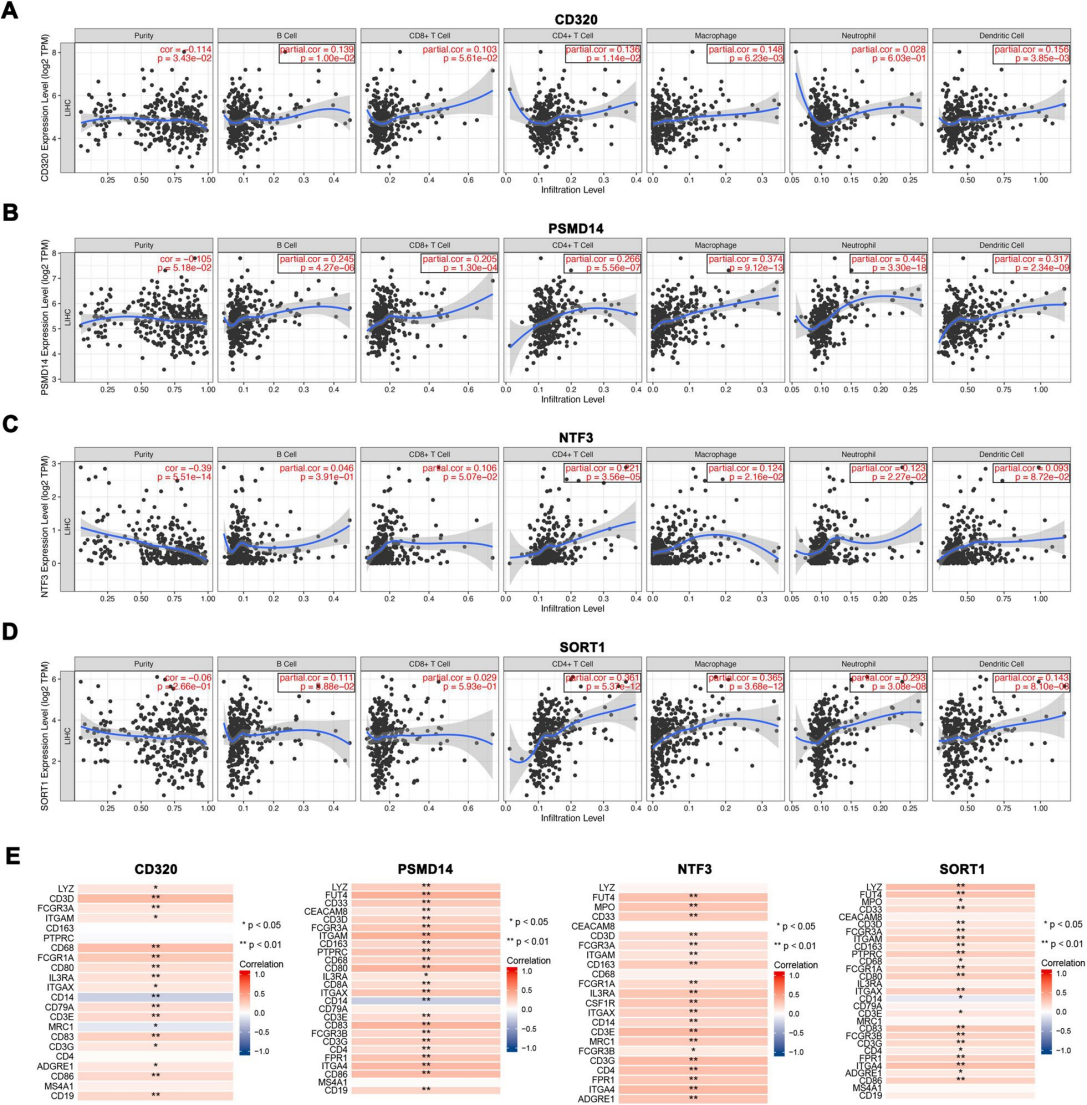
Correlation between miRNA targeted-genes expression and immune infiltration. **A** Correlation of CD320 expression with immune infiltration level in HCC. **B** Correlation of PSMD14 expression with immune infiltration level in HCC. **C** Correlation of NTF3 expression with immune infiltration level in HCC. **D** Correlation of SORT1 expression with immune infiltration level in HCC. **E** The correlation between the four hub genes (CD320, PSMD14, SORT1, and NTF3) and the cell markers of corresponding immune cells by Cell Marker database

We further investigated whether certain links exist between genes and immune cells. The correlations were explored from the Tumor Immune Estimation Resource (TIMER) database, which showed that CD320 was positively associated with B cells, CD4+ T cells, macrophages, and dendritic cells immune infiltration level (Fig. 5A); PSMD14 was positively associated with B cells, CD8+ T cells, CD4+ T cells, macrophages, neutrophils, and dendritic cell infiltration (Fig. 5B); NTF3 was negatively associated with tumor purity and was related to CD4+ T cells, macrophage, and neutrophil infiltration (Fig. 5C), and SORT1 was positively associated with B cells, CD4+ T cells, macrophages, neutrophils, and dendritic cells immune infiltration level (Fig. 5D). Meanwhile, the average expression heatmaps of CD320, SORT1, NTF3, and PSMD14 in various immune cells are shown in Additional file 1: Fig. S5 using the TISCH database.

Based on the above results, we further investigated the correlation between these four hub genes and the cell markers of the corresponding immune cells. Immune marker data were obtained from the Cell Marker database (http://bio-bigdata.hrbmu.edu.cn/CellMarker/). Correlation heatmaps are presented in Fig. 4E.

### Relationship between miRNA targeted-genes expression and methylation

**Fig. 6:**
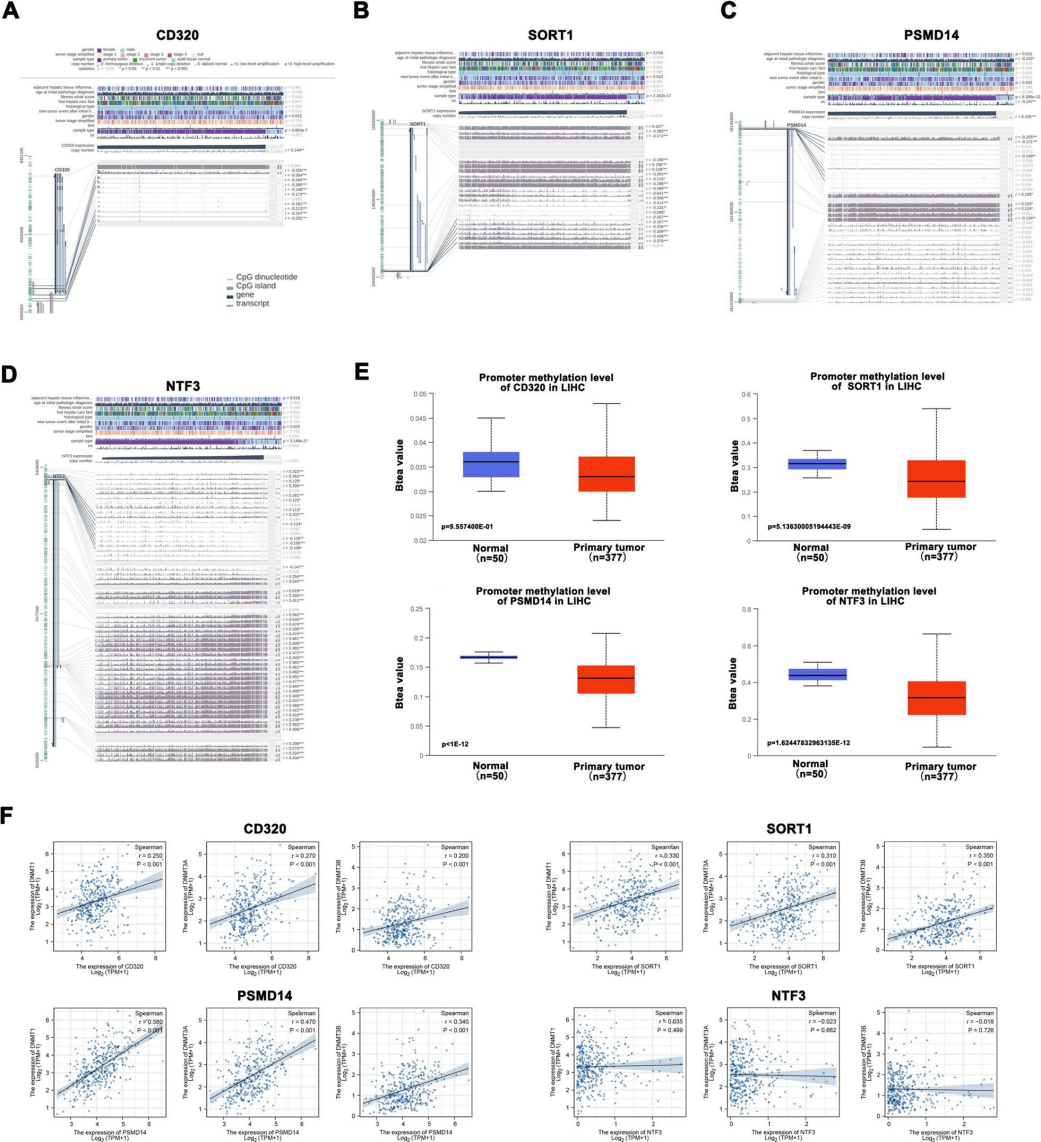
Relationship between miRNA targeted-genes expression and methylation. **A**–**E** Correlation between gene expression (CD320, PSMD14, SORT1, and NTF3) and promoter methylation levels. **F** The correlation analyses between the four hub genes (CD320, PSMD14, SORT1, and NTF3) and related methyltransferase genes (DNMT1, DNMT3A, and DNMT3B)

Previous studies reported that tumor purity as a confounding factor affects gene expression and DNA methylation levels, and copy number affects gene expression levels, which in turn is related to tumor purity and immune cell infiltration levels [20]. Therefore, we obtained the methylation expression levels of four genes from the UALCAN database. We also in-depth investigated the relationship between gene expression and methylation levels in HCC using the MEXPRESS database. We found that the promoter methylation level of CD320 in HCC tissues was significantly lower than that in normal tissues, and CD320 expression was negatively related to its promoter methylation level (Fig. 6A, E). Moreover, similar to CD320, the promoter methylation levels of SORT1 and PSMD14 were considerably lower in tumor tissues than in normal tissues, and there was also a negative correlation between gene expression and promoter methylation levels (Fig. 6B, C, E). However, the analysis results of NTF3 differed from those of CD320, SORT1, and PSMD14. NTF3 expression was positively associated with promoter methylation levels (Fig. 6D, E). This partly indicates that NTF3 as a tumor suppressor gene and SORT1, CD320, and PSMD14 as oncogenes regulate HCC progression. Next, we performed correlation analyses between the four genes and related methyltransferase genes (DNMT1, DNMT3A, and DNMT3B). The results showed that HCC tumors with high levels of CD320, SORT1, and PSMD14 had high levels of methyltransferase genes (DNMT1, DNMT3A, and DNMT3B), whereas there was no significant correlation between NTF3 and methyltransferase genes (Fig. 6F). Moreover, we analyzed the relationship between miRNA (hsa-miR-21-5p and hsa-miR125b-5p) and methyltransferase genes (DNMT1, DNMT3A, and DNMT3B) in HCC. As shown in Additional file 1: Fig. S6A, B, the results showed that there was a positive relationship between hsa-miR-21-5p and DNMT1 (R = 0.190, *p* < 0.001) and DNMT3A (R = 0.170, *p* < 0.001), but there was no significant relationship between hsa-miR-21-5p and DNMT3B (R = 0.013, *p* = 0.811). Furthermore, a significant negative relationship was observed between hsa-miR125b-5p and DNMT1 (R = − 0.270, *p* < 0.001), DNMT3A (R = − 0.400, *p* < 0.001), and DNMT3B (R = − 0.190, *p* < 0.001). Together, these results provide important insights that altered methylation levels may contribute to the function of these hub genes.

### Gene set enrichment analysis of miRNA targeted-genes in HCC tissues

**Fig. 7:**
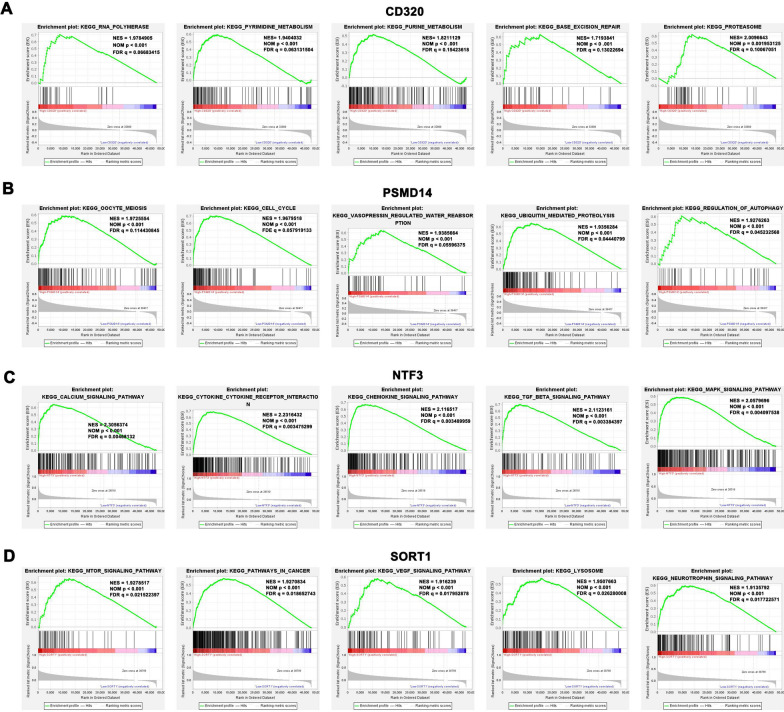
Gene set enrichment analysis of miRNA targeted-genes in HCC tissues. **A**–**D** The GSEA analysis of CD320, PSMD14, NTF3, and SORT1

To study the downstream pathways of these miRNA-targeted genes, we grouped the matrix of TCGA database according to the gene expression level for GSEA analysis. We chose five of all statistically significant analysis results. CD320 is related to RNA Polymerase (NES = 1.9784905, NOM *p* < 0.001, FDR = 0.06683415), Pyrimedine Metabolism (NES = 1.9404032, NOM *p* < 0.001, FDR = 0.063131504), Purine Metabolism (NES = 1.8211129, NOM *p* < 0 0.001, FDR = 0.15423618), Base Excision Repair (NES = 1.7193841, NOM *p* < 0 0.001, FDR = 0.13022694), and Proteasome (NES = 2.0096643, NOM *p* = 0.001953125, FDR = 0.10067051) (Fig. 7A). PSMD14 was associated with oocyte meiosis (NES = 1.9725554, NOM *p* < 0.001, FDR = 0.114430845), cell cycle (NES = 1.9679518, NOM *p* < 0.001, FDE = 0.057919133), vasopressin-regulated water reabsorption (NES = 1.9385664, NOM *p* < 0.001, FDR = 0.05596375), ubiquitin-mediated proteolysis (NES = 1.9356284, NOM *p* < 0.001, FDR = 0.04440799), and regulation of autophagy (NES = 1.9276263, NOM *p* < 0.001, FDR = 0.045232568) (Fig. 7B). NTF3 was associated with the calcium signaling pathway (NES = 2.3056374, NOM *p* < 0.001, FDR = 0.00468132), cytokine-cytokine receptor interaction (NES = 2.2316432, NOM *p* < 0.001, FDR = 0.003475299), chemokine signaling pathway (NES = 2.116517, NOM *p* < 0.001, FDR = 0.003489959), TGF-β signaling pathway (NES = 2.1123161, NOM *p* < 0.001, FDR = 0.003384397), and MAPK signaling pathway (NES = 2.0579696, NOM *p* < 0.001, FDR = 0.004097538) (Fig. 7C). SORT1 was associated with the mTOR signaling pathway (NES = 1.9278517, NOM *p* < 0.001, FDR = 0.021522397), pathways in cancer (NES = 1.9270834, NOM *p* < 0.001, FDR = 0.018652743), VEGF signaling pathway (NES = 1.916239, NOM *p* < 0.001, FDR = 0.017952878), lysosome (NES = 1.9507663, NOM *p* < 0.001, FDR = 0.026280008), and neurotrophin signaling pathway (NES = 1.9135792, NOM *p* < 0.001, FDR = 0.017722571) (Fig. 7D).

### Survival analysis and prognostic model

**Fig. 8:**
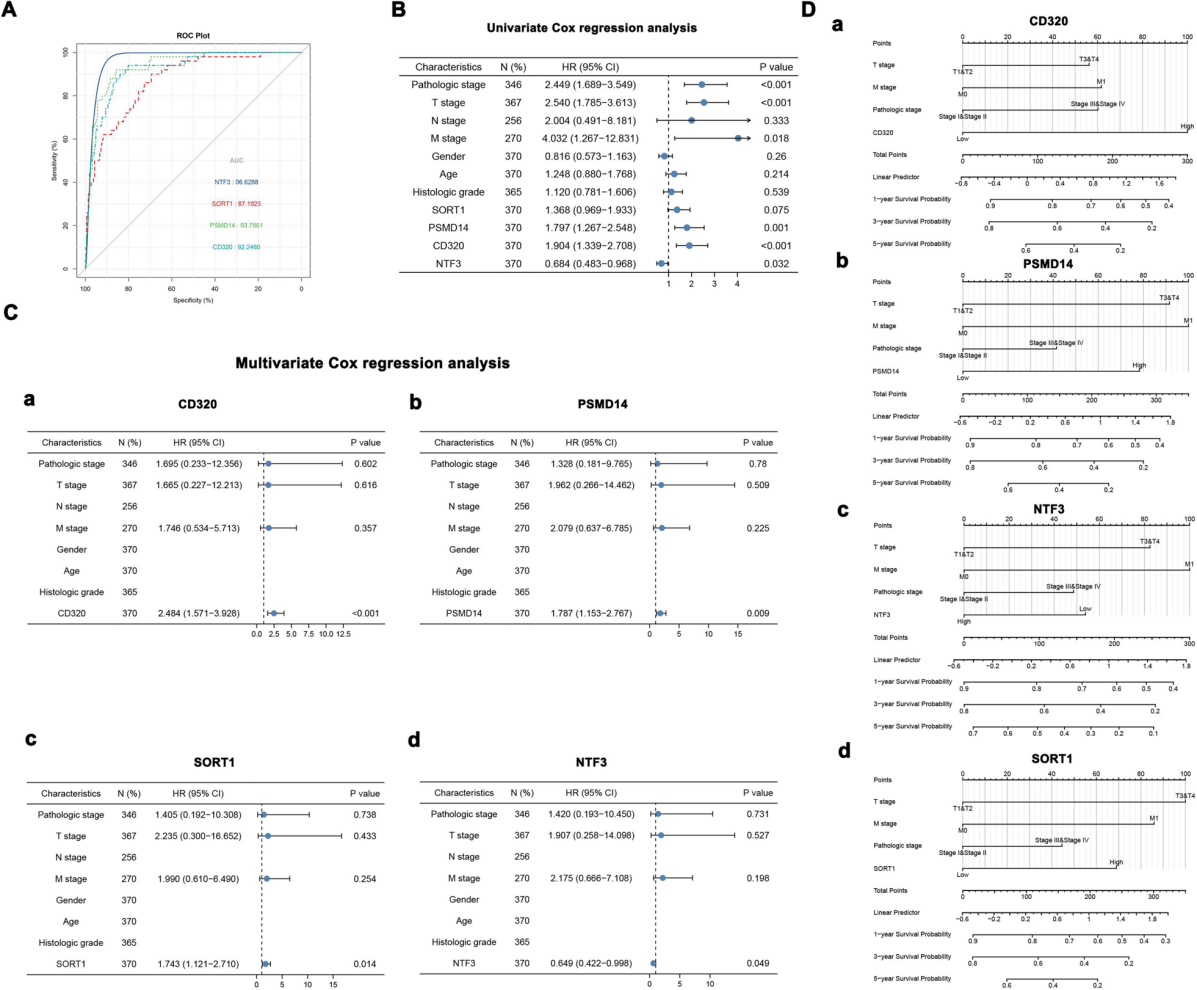
Survival Analysis and Prognostic Model. **A** The ROC curve of CD320, PSMD14, NTF3, and SORT1 in HCC. **B** The univariate cox regression analysis of CD320, PSMD14, NTF3, SORT1 and clinicopathological characteristics. **C** The multivariate cox regression analysis of CD320, PSMD14, NTF3, SORT1. **D** The Nomogram shows the prognostic model of CD320, PSMD14, NTF3, SORT1

To investigate the impact of four key genes on the prognosis of HCC, an effective model was established for predicting prognostic status by univariate and multivariate Cox proportional hazards regression analysis. The area under the curve (AUC) of the ROC curve showed that CD320 (AUC = 0.922460), PSMD14 (AUC = 0.937861), SORT1 (AUC = 0.871925), and NTF3 (AUC = 0.966288) were significant predictors (Fig. 8A). In the univariate Cox proportional hazards regression analysis, four genes and tumor stage were identified as prognostic biomarkers (Fig. 8B). Multivariate Cox proportional hazards regression analysis showed that CD320 (HR = 2.484, 95% CI = 1.571–3.928, *p* < 0.001), PSMD14 (HR = 1.787, 95% CI = 1.153–2.767, *p* = 0.009), and SORT1 (HR = 1.743, 95% CI = 1.121–2.710, *p* = 0.014), with significant effects on prognosis, were identified (Fig. 8C-a–c). Meanwhile, NTF3 (HR = 0.649, 95% CI = 0.422–0.998, *p* = 0.049) was also statistically significant in the multivariate Cox proportional hazards regression analysis (Fig. 8C-d). A nomogram was used for prognostic judgment. “Points” is a scoring scale for each factor, and “Total points” is a scale for total score (Fig. 8D-a–d). Moreover, the results of the calibration analysis suggest that the four prognostic models were in good concordance with the outcomes of HCC patients (Additional file 1: Fig. S7A–D).

### Validation of miRNA and their targeted genes expressions

**Fig. 9:**
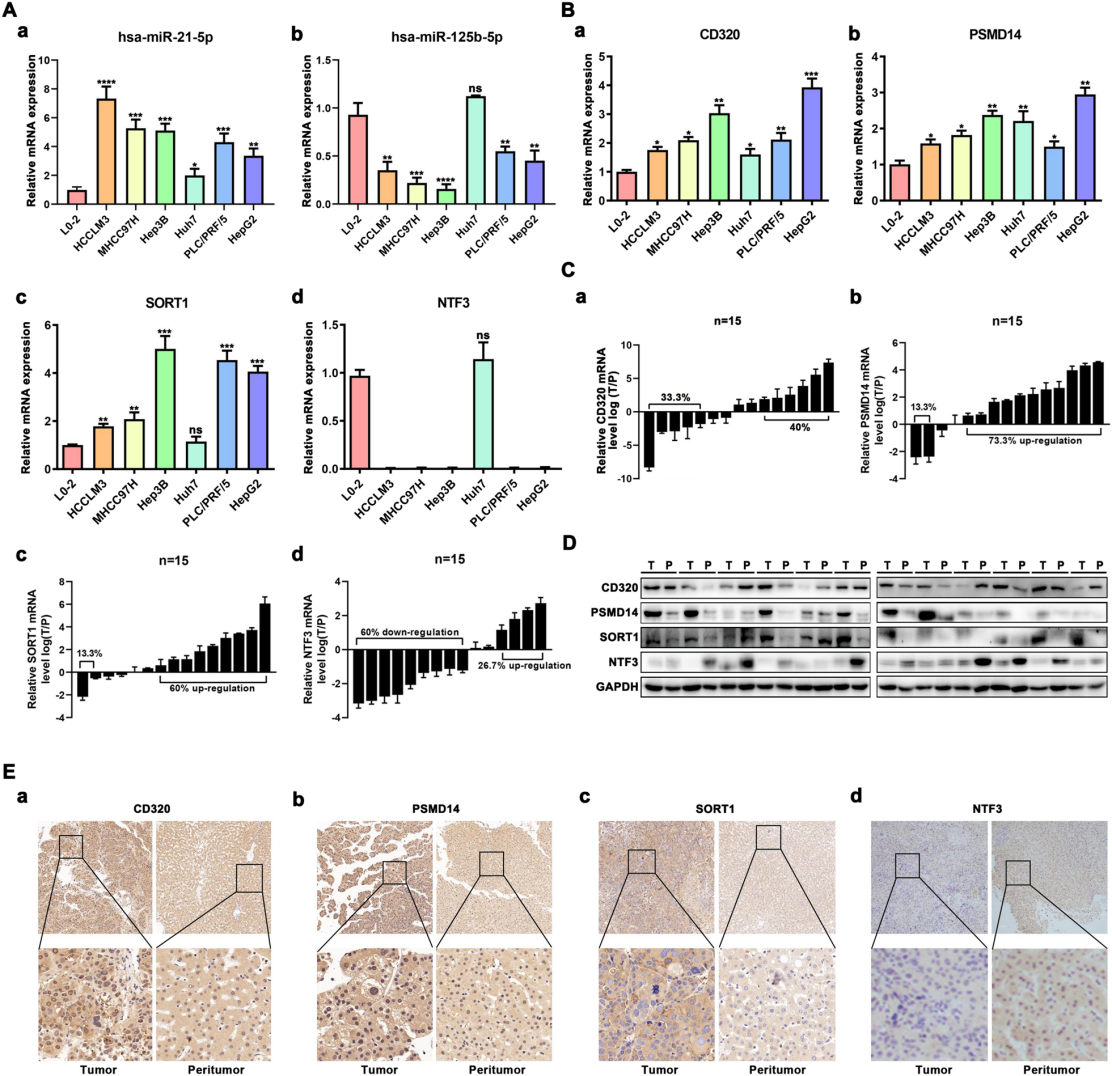
Validation of miRNA and their targeted-genes expressions. **A**-**a**, **b** and **B**-**a**–**d** The qRT-PCR results of hsa-miR-125b-5p, hsa-miR-21-5p, CD320, PSMD14, SORT1, and NTF3 in different cell lines. **C-a**–**d** The qRT-PCR results of CD320, NTF3, PSMD14 and SORT1 in 15 pairs of human liver cancer and para-cancerous tissues. **D** Western blot of CD320, NTF3, PSMD14 and SORT1 in 12 pairs HCC tumor tissues and matched adjacent normal tissues. **E-a**–**d** IHC of CD320, NTF3, PSMD14 and SORT1 in 15 pairs HCC tumor tissues and matched adjacent normal tissues. (*P < 0.05, **P < 0.01, ***P < 0.001, ****P < 0.0001, n.s. not statistically significant)

We validated the expression of two miRNAs and their target genes in HCC cell lines and HCC samples using qRT-PCR. hsa-miR-21-5p expression was significantly upregulated in six HCC cell lines (HCCLM3, MHCC97H, Hep3B, Huh7, PLC/PRF/5, and HepG2) compared to that in L02 cells (Fig. 9A-a). In contrast, hsa-miR-125b-5p expression was significantly upregulated in L02 liver cells compared to that in the five HCC cell lines (Fig. 9A-b). The mRNA expression of CD320 and PSMD14 was significantly upregulated in six HCC cell lines (HCCLM3, MHCC97H, Hep3B, Huh7, PLC/PRF/5, and HepG2) compared to liver cells L02 (Fig. 9B-a, b). The mRNA level of SORT1 was higher in the five HCC cell lines than in the L02 cells (Fig. 9B-c). In contrast, NTF3 mRNA levels in five HCC cell lines (HCCLM3, MHCC97H, Hep3B, PLC/PRF/5, and HepG2), except for huh7 cell lines, were remarkably lower than those in liver cells L02 (Fig. 9B-d). Interestingly, we found that the expression of miRNAs and mRNAs did not significantly change in Huh7 cells. Then, we selected 15 pairs of human liver cancer and para-cancerous tissues to further detect the expression levels of the four hub genes. The results showed that NTF3 was downregulated in 9 of 15 HCC tissues, PSMD14 was upregulated in 11 of 15 HCC tissues, and SORT1 was upregulated in 9 of 15 HCC tissues (Fig. 9C-a–c).

CD320 expression was not significantly different between HCC tumor tissues and matched adjacent normal tissues (Fig. 9C-d). Next, western blot analysis was used to validate the protein levels of PSMD14, SORT1, and NTF3 in 12 pairs of HCC tissues and normal liver tissues (Fig. 9D). Moreover, we further explored the changes of 4 genes protein expression from the Human Protein Atlas database. The results indicated that CD320, PSMD14, and SORT1 protein levels were higher in tumor tissues than in normal tissues (Additional file 1: Fig. S8A, B, D), while the level of NTF3 in the normal tissues was higher than that in the tumor tissues (Additional file 1: Fig. S8C). Similarly, immunohistochemistry assay indicated that the levels of CD320, PSMD14, and SORT1 were higher than those in matched adjacent normal tissues in 15 HCC patients (Fig. 9E-a–c), whereas NTF3 showed the opposite result (Fig. 9E-d). We also utilized the GSE14520, GSE76427, and TCGA expression matrices to investigate the expression levels of the four hub genes (Additional file 1: Fig. S9A–C).

### Validation of the potential mechanisms in HCC

**Fig. 10:**
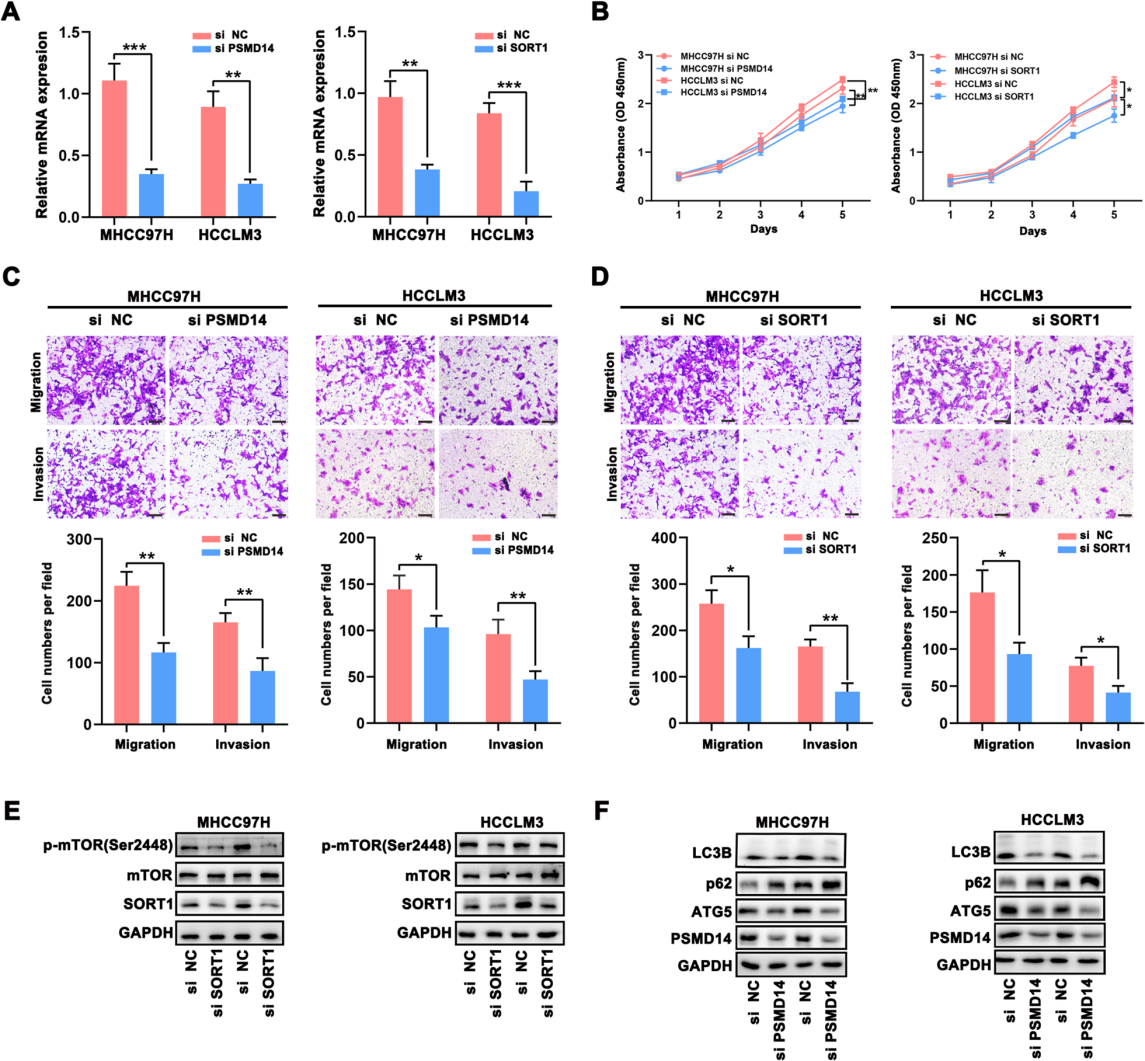
Validation of the potential mechanisms in HCC. **A** The interference of PSMD14 and SORT1 were confirmed using qRT-PCR. **B** The proliferation of HCC cells was assessed using CCK-8 assay. **C**, **D** The migration and invasion of HCC cells assessed by transwell assay. **E**, **F** The protein levels of mTOR pathway and autophagy-related biomarkers in HCC cells with si-SORT1 and si-PSMD14 were detected using western blot

According to the previous PCR, MHCC97H and HCCLM3 cell were selected for the further experiments. The PSMD14 and SORT1 expression in MHCC97H and HCCLM3 cells transfected with siRNA were confirmed using qRT-PCR (Fig. 10A). And the CCK-8 and transwell assays suggest that the downregulation of PSMD14 and SORT1 can slow down the growth and migration and invasion of MHCC97H and HCCLM3 cells (Fig. 10B–D). Furthermore, the results of cellular function assay are consistent with recent studies, suggesting that the oncogenic role of PSMD14 and SORT1 in various cancer [21–24]. However, the potential mechanisms of them in HCC are still unclear. Above GSEA reveals that PSMD14 is related to the autophagy process and SORT1 is association with the mTOR signaling pathway. Therefore, we detected the protein expression levels of these pathways’ biomarker by performing the western blot assay. And we found that the interference of SORT1 downregulates the p-mTOR (Ser2448) expression, leading to the inactivation of mTOR signaling pathway (Fig. 10E). Then, the autophagy process in HCC cells was inhibited after the downregulation of PSMD14. The LC3B and ATG5 expression were significantly decreased and p62 protein was remarkably increased when interfering the PSMD14 (Fig. 10F). Consistent with GSEA, SORT1 can activate the mTOR pathway to enhance the HCC progression. Meanwhile, we conjectured that elevated PSMD14 may maintain tumor cell survival by stimulating autophagy enabling then to ensure their own energy metabolism as well as reduce damage under specific circumstances. All in all, these results reveal the oncogenic role of SORT1 and PSMD14 in HCC cells.

### Prediction of DEIRGs-related drugs

The miRNA targeted genes screened before were uploaded to DSigDB for drug prediction enrichment analysis (Additional file 1: Table S1). We then chose the top 10 drugs that were related to miRNA-targeted genes. The screen criteria were adjusted with p-value < 0.05. The results of the drug prediction enrichment analysis are shown in Additional file 1: Fig. S10 and Table S1. We found that pinaflavol TTD 00010236, harmaline CTD 00006074, GNF-Pf-3464 TTD 00008265, sulfuretin TTD 00011132, chloroxine TTD 00007143, Tyrphostin B48 CTD 00003485, chloroxine, and Redoxal TTD 00010526 may be associated with PSMD14; AlphaRedisol BOSS may be associated with CD320, and selenium methyl cysteine CTD 00000103 may be associated with SORT1. Some of these drugs have been reported to have anti-cancer effects. For example, harmaline can suppress the growth of liver cancer cells by inducing the p53/p21 and Fas/FasL signaling pathways [25], and may have therapeutic potential for controlling breast cancer invasiveness [26], and chloroxine can facilitate platinum-induced DNA damage to induce cancer cell death in high-grade serous cancer [27]. Therefore, the prediction of DERIGs-related drugs as a good reference advances our future scientific research.

## Discussion

The ceRNA regulatory network is thought to play a role in carcinogenesis, according to earlier research [28, 29], including breast cancer [30], lung cancer [31, 32], gastric cancer [33, 34], and pancreatic cancer [35]. However, few studies have focused on a comprehensive ceRNA regulatory network, which is linked to immune filtration for predicting the prognosis of HCC. Accordingly, in this study, we attempted to validate some promising biomarkers via the miRNA-mRNA interaction mechanism, which are related to immune cells.

In our study, we first identified 9 DEMis, 97 upregulated, and 37 downregulated differentially expressed immune-related mRNAs. Next, we chose the top 20 up-/down-regulated mRNAs for further functional enrichment analysis, and the results showed that the top 20 upregulated and downregulated DEIRGs were associated with the MAPK signaling pathway, cytokine-cytokine receptor interaction, and PI3K-AKT signaling pathway. Key DEGs such as CD320, PSMD14, NTF3, and SORT1 were identified as key genes according to survival analysis, and the miRNA-mRNA network revealed that hsa-miR-125b-5p and hsa-miR-21-5p may act as sponges of the four key immune-related DEGs (CD320, PSMD14, NTF3, and SORT1).

Previous studies have suggested that immune infiltration affects patient prognosis [36, 37]. Therefore, we also explored the correlation between immune infiltration and the four prognostic DEMs using the TIMER and TISCH databases. In this study, we found that CD320, PSMD14, NTF3, and SORT1 were associated with some types of immunocytes. B cells, CD4+ T cells, macrophages, and dendritic cells were positively correlated with CD320; PSMD14 was positively correlated with B cells, CD8+ T cells, CD4+ T cells, neutrophils, and dendritic cells; NTF3 was negatively related to tumor purity and positively associated with CD4+ T cells and neutrophils, and SORT1 is positively associated with CD4+ T cells, macrophages, and neutrophils. Moreover, it was reported that DNA methylation as a confounding factor affects tumor purity and the infiltration levels of immunocytes [20]. Therefore, the methylation levels of these genes may also be important factors leading to disease. Our study found that CD320, PSMD14, NTF3, and SORT1 expression are associated with promoter methylation levels through the MEXPRESS and UALCAN databases. More importantly, the correlation analysis also showed that there is a close relationship between altered methylation levels and gene expression.

In addition, the GSEA results of multiple genes showed that they were highly enriched in cytokine-cytokine receptor interactions and chemokine signaling pathways. Then, an effective model was established for predicting the prognostic status using univariate and multivariate Cox proportional hazards regression analyses. The four prognostic immune-related DEMs and clinicopathological features were validated and found to be independent prognostic factors for HCC. The calibration curve of the model also showed a good prediction function for prognosis. In addition, the results of qRT-PCR, immunohistochemistry, and western blot were consistent with our bioinformatics results.

The deubiquitinating enzyme (DUB) 26S proteasome non-ATPase regulatory subunit 14 (PSMD14, also known as RPN11 and POH1) is a component of the 19S regulatory cap in the 26S proteasome, which belongs to the JAB1/MPN/Mov34 (JAMM) domain [38]. Previous studies have reported that PSMD14 is involved in a variety of biological processes, including cell viability [39, 40], double-strand DNA break repair [41, 42], cell differentiation [40, 43, 44], and tumor progression [45] by regulating protein deubiquitination and stabilization [46]. Furthermore, the high expression of PSMD14 in several cancers has been validated and reported to act as an oncogene in several human cancers. For instance, PSMD14 is upregulated in esophageal squamous cell carcinoma (ESCC) tissues and can promote tumor cell migration and invasion through the PSMD14/SNAIL axis [47]. Similarly, in head and neck squamous cell carcinoma (HNSCC), PSMD14 decreased E2F1 ubiquitination and degradation, which improved AKT pathway activation and SOX2 transcription, thereby facilitating HNSCC growth, chemoresistance, and stemness [48]. Additionally, PSMD14 can accelerate hepatocellular carcinoma development and metastasis by stabilizing GRB2 [22]. However, other mechanisms of PSMD14-mediated tumor progression, such as the immune microenvironment, remain elusive.

NTF3 is a member of the nerve growth factor (NGF) family [49] and plays a critical role in neuronal differentiation, survival, neurite growth, and neurotransmitter synthesis by binding Trk receptors (high affinity) and receptor p75NTR (low affinity) [50–52]. Furthermore, NTFs have been shown to contribute to tumor progression in a variety of cancers, including testicular germ cell tumors (TGCTs) [53], human hepatocellular carcinoma (HCC) [54], intrahepatic cholangiocarcinoma (ICC) [55], and breast cancer [56].

Although this study is the first to investigate miRNA-mRNA interactions that are closely correlated with the infiltration of immunocytes by multiple databases, there are still some limitations. First, we only compared the tumor tissues with normal tissues in HCC. Key miRNAs and genes in different periods, such as metastatic HCC, require further exploration. Second, although we identified abnormal miRNAs (hsa-miR-125b-5p and hsa-miR-21-5p) and their target genes (NTF3, PSMD14, CD320, and SORT1), which might be prognostic predictors for HCC using TCGA data, GEO data, and other databases, we also validated gene expression in normal liver cells and HCC cell lines by qRT-PCR, immunohistochemistry, and western blot. Functional experiments in vitro and animal models in vivo should be added in further studies. Third, the source of the microarrays is only the tissues. Body fluid-like serum may contain circulating miRNAs, which are more likely to be accepted for clinical application.

For further experiments, we found that SORT1 is close to mTOR signaling pathway, which lead to the tumor progression. According to previous studies, the role of mTOR in cancers has been well investigated. Also, several mTOR-related drugs have been developed for the treatment of cancers before [57, 58]. So our experimental finding that SORT1 can activate the mTOR signaling pathway to cause tumorigenesis is very promising, and the subsequent in-depth study may achieve unexpected results. However, autophagy-related studies are still somewhat controversial. Initial studies found that autophagy inhibits tumorigenesis [59]. Moreover, it has been found that tumors can also maintain their own survival with the help of autophagy. Yang et al. found that some tumor cell lines maintain abnormal high levels of autophagy even under energetic conditions, probably because the intense metabolic stress forces the cells to increase their autophagy levels to keep their survival [60, 61]. In our study, we also found that the PSMD14 is related to the autophagy process in HCC cells. The experiments show that PSMD14 may maintain the HCC cell survival by inducing the autophagy process. However, there are still some limitation in our study. We are temporarily unable to investigate the mechanisms of all genes, but the current study is considered to provide some help in the current field and serve as a good reference for subsequent studies.

## Conclusion

In conclusion, six potential immune-related prognostic predictors or biomarkers including two abnormal miRNAs (hsa-miR-125b-5p and hsa-miR-21-5p) and four targeted genes (NTF3, PSMD14, CD320, and SORT1) were identified in HCC, which may be closely correlated with the infiltration of immunocytes. Meanwhile, further experiments found that SORT1-mediated activation of mTOR pathway and PSMD14-mediated autophagy process may induce the progression of HCC, which indicates that our study as a good reference for future studies can accelerate the development of HCC-related biomarkers and treatments.

## Supplementary Information

**Additional file 1: Figure S1.** Quantile normalization of miRNA microarray data (GSE69580) containing five tumors and five normal tissues. **Figure S2.** Overlapping genes between miRNA (seven upregulated and two downregulated miRNAs) target genes and immune genes from the ImmPort Portal database. **Figure S3.** The normalization of 393 genes from the TCGA database. **Figure S4.** (A) Expression levels and (B) overall survival rate of 11 genes obtained from the TCGA database. (*P < 0.05, **P < 0.01, ***P < 0.001, ****P < 0.0001, n.s. not statistically significant). **Figure S5.** The heatmap of CD320, PSMD14, NTF3, and SORT1 expression in different immune cells. **Figure S6.** The correlation analyses between (A) miR-21-5p, (B) miR-125b-5p and related methyltransferase genes (DNMT1, DNMT3A, and DNMT3B). **Figure S7.** (A–D) The calibration analysis of the 4 prognostic nomogram models (CD320, PSMD14, SORT1, and NTF3). **Figure S8.** (A–D) Immunohistochemistry validation of gene protein expression (CD320, PSMD14, NTF3, and SORT1) from the Human Protein Atlas database. **Figure S9.** Investigate the 4 hub genes (CD320, PSMD14, SORT1, and NTF3) expression level in the GSE14520 (T = 247; N = 241) (A), GSE76427 (T = 115; N = 52) (B), and TCGA (T = 371; N = 160) (C) expression matrix. (*P < 0.05, **P < 0.01, ***P < 0.001, ****P < 0.0001, n.s. not statistically significant). **Figure S10.** Prediction of DEIRGs-related drugs. **Table S1.** Prediction of DEIRGs-related top 10 drugs.

## Data Availability

The datasets used and/or analyzed during the current study are available in the Figshare (DOI: 10.6084/m9.figshare.17105165).

## Abbreviations

HCC: Hepatocellular carcinoma
TCGA: The Cancer Genome Atlas
HPA: The Human Protein Atlas
GEO: The Gene Expression Omnibus
DEmiRNAs, DEmRNAs: Differentially expressed miRNAs/mRNAs
DEIRGs: Differentially expressed immune-related genes
GO: Gene Ontology
KEGG: Kyoto Encyclopedia of Genes and Genomes
