# Supplementary material for: Six immune-related promising biomarkers may promote hepatocellular carcinoma prognosis: a bioinformatics analysis and experimental validation
Source: Cancer Cell Int. 2023 Mar 23;23:52. doi: 10.1186/s12935-023-02888-9 (PMC10035283; doi:10.1186/s12935-023-02888-9)
Supplement: Supplementary file 1 — Additional file 1: Figure S1. Quantile normalization of miRNA microarray data (GSE69580) containing five tumors and five normal tissues. Figure S2. Overlapping genes between miRNA (seven upregulated and two downregulated miRNAs) target genes and immune genes from the ImmPort Portal database. Figure S3. The normalization of 393 genes from the TCGA database. Figure S4. (A) Expression levels and (B) overall survival rate of 11 genes obtained from the TCGA database. (*P < 0.05, **P < 0.01, ***P < 0.001, ****P < 0.0001, n.s. not statistically significant). Figure S5. The heatmap of CD320, PSMD14, NTF3, and SORT1 expression in different immune cells. Figure S6. The correlation analyses between (A) miR-21-5p, (B) miR-125b-5p and related methyltransferase genes (DNMT1, DNMT3A, and DNMT3B). Figure S7. (A–D) The calibration analysis of the 4 prognostic nomogram models (CD320, PSMD14, SORT1, and NTF3). Figure S8. (A–D) Immunohistochemistry validation of gene protein expression (CD320, PSMD14, NTF3, and SORT1) from the Human Protein Atlas database. Figure S9. Investigate the 4 hub genes (CD320, PSMD14, SORT1, and NTF3) expression level in the GSE14520 (T = 247; N = 241) (A), GSE76427 (T = 115; N = 52) (B), and TCGA (T = 371; N = 160) (C) expression matrix. (*P < 0.05, **P < 0.01, ***P < 0.001, ****P < 0.0001, n.s. not statistically significant). Figure S10. Prediction of DEIRGs-related drugs. Table S1. Prediction of DEIRGs-related top 10 drugs. [file 12935_2023_2888_MOESM1_ESM.docx]

Additional file 1

# Additional Figures and Tables

## Additional Figures

##
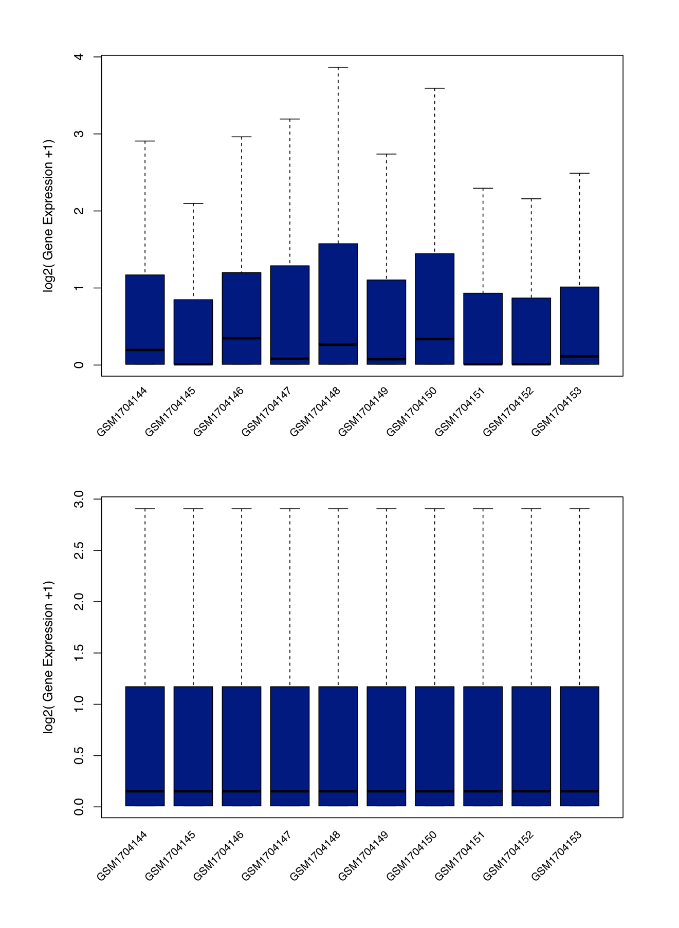


## Figure S1. Quantile normalization of miRNA microarray data (GSE69580) containing five tumors and five normal tissues.


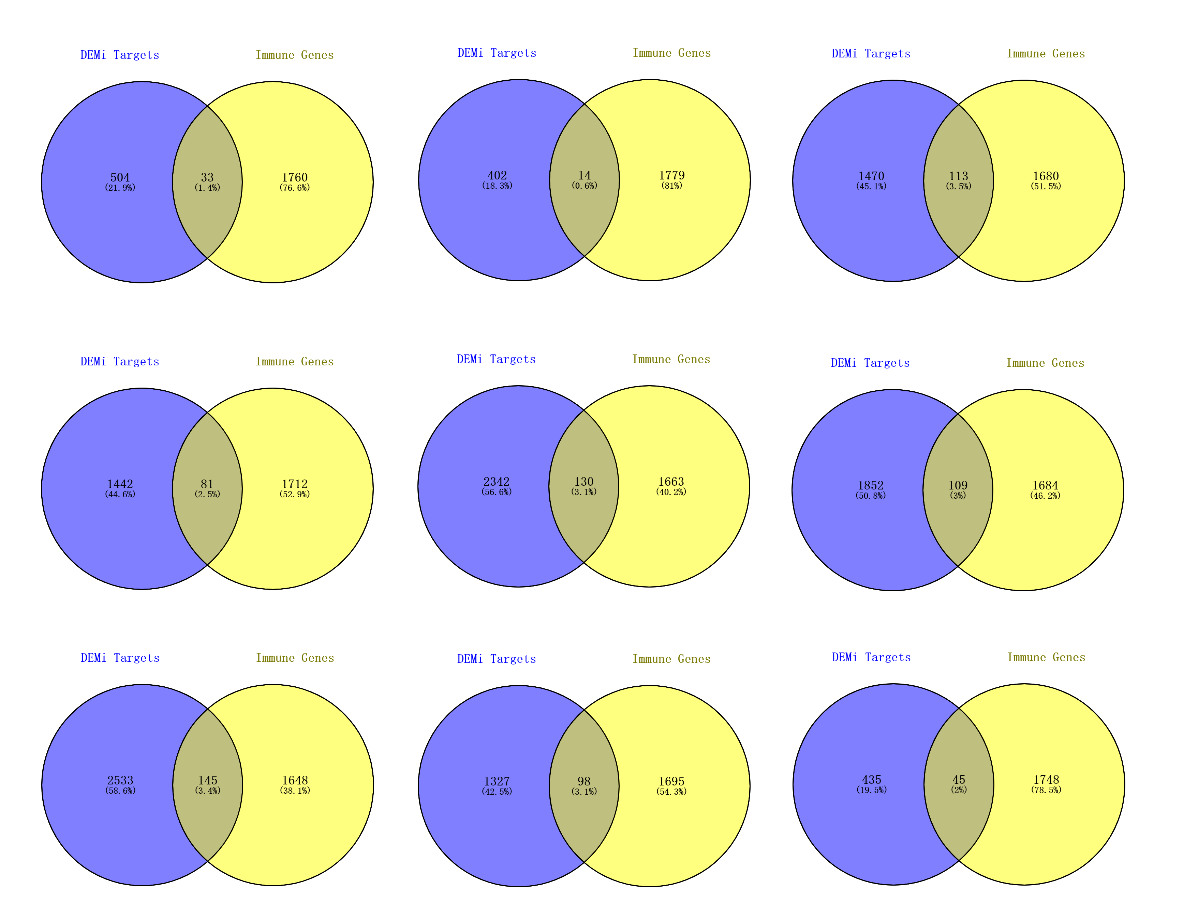


## Figure S2. Overlapping genes between miRNA (seven upregulated and two downregulated miRNAs) target genes and immune genes from the ImmPort Portal database.


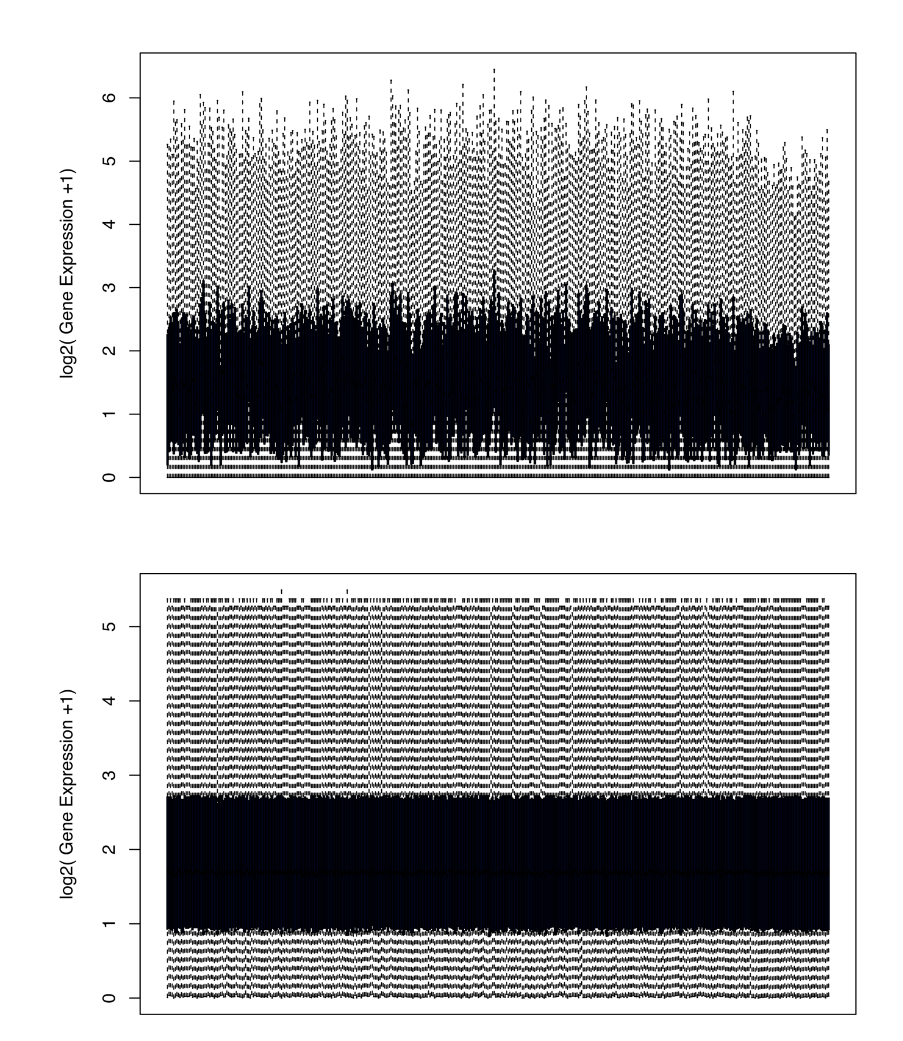


## Figure S3. The normalization of 393 genes from the TCGA database.


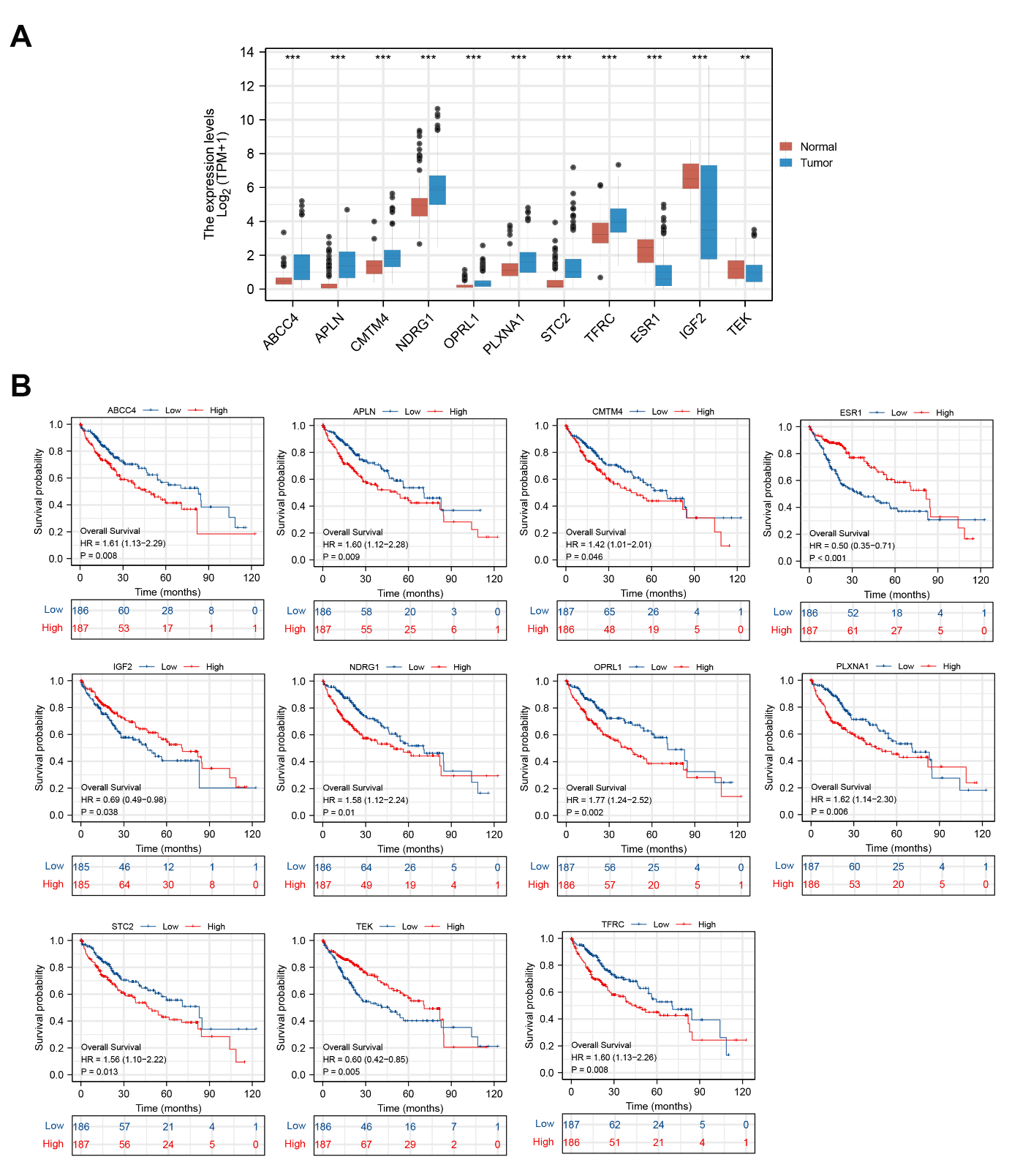


**Figure S4**. **(A)** Expression levels and **(B)** overall survival rate of 11 genes obtained from the TCGA database. (*P<0.05, **P<0.01, ***P<0.001, ****P<0.0001, n.s. not statistically significant)


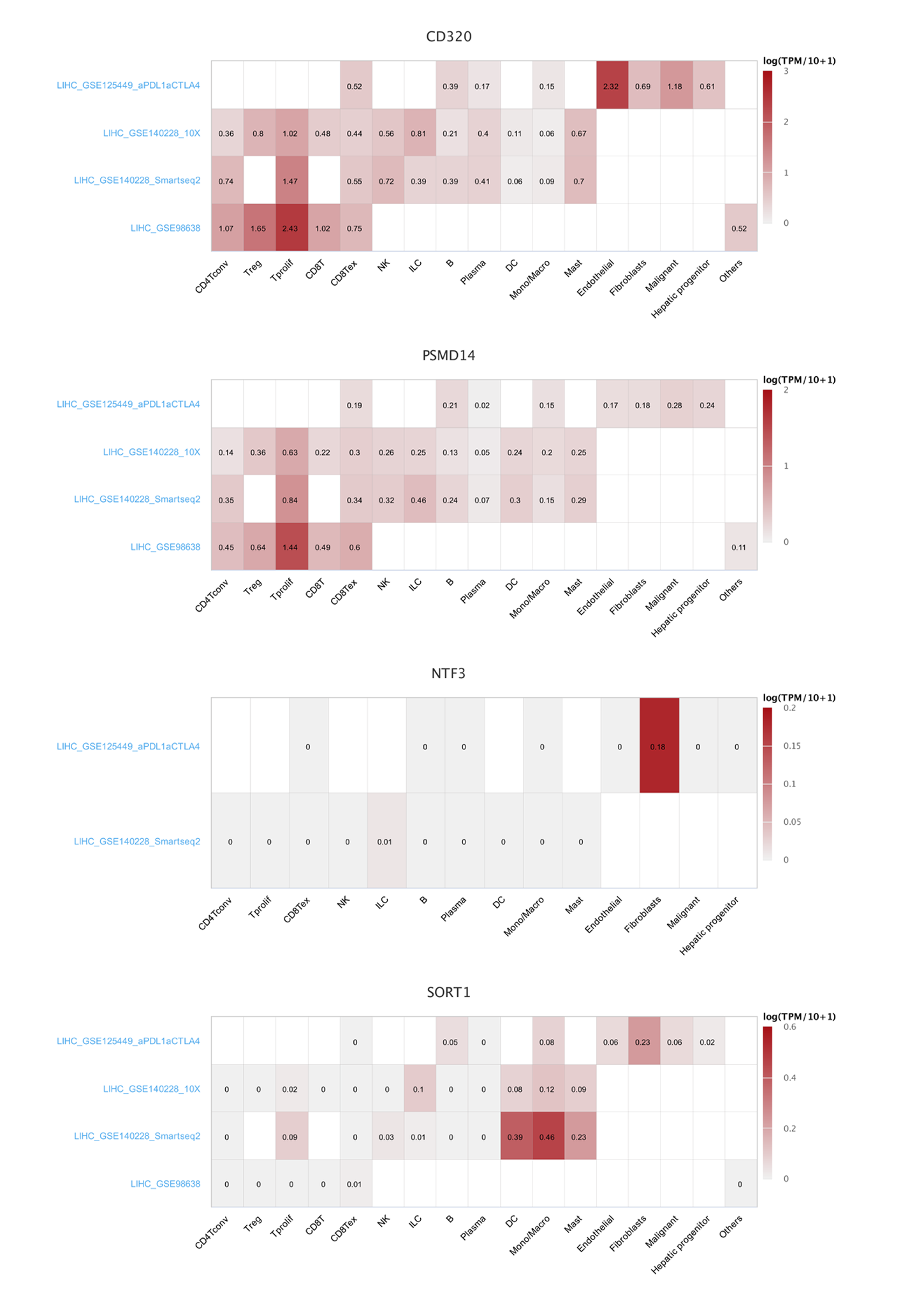


**Figure S5**. The heatmap of CD320, PSMD14, NTF3, and SORT1 expression in different immune cells.


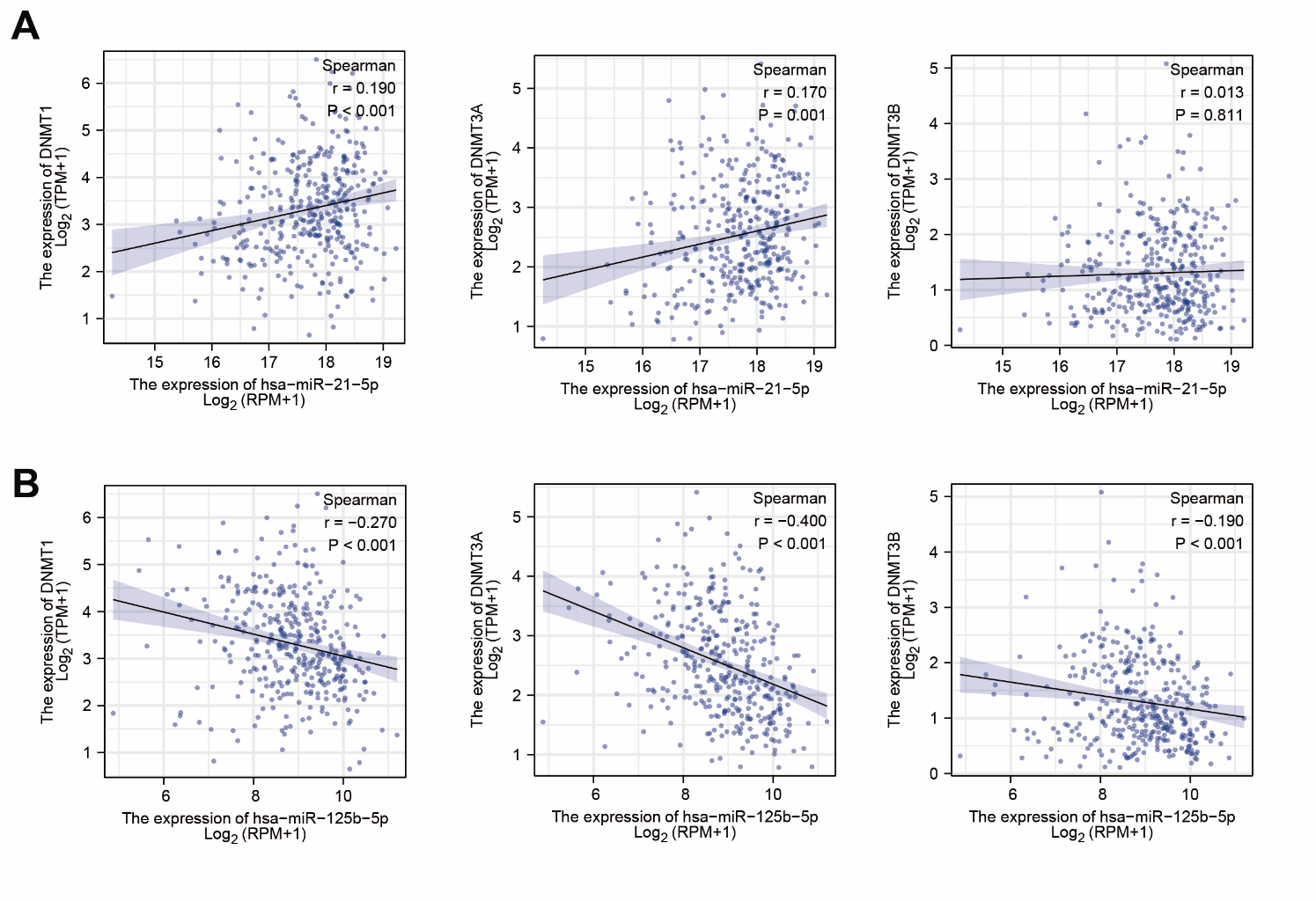


**Figure S6** The correlation analyses between **(A)** miR-21-5p, **(B)** miR-125b-5p and related methyltransferase genes (DNMT1, DNMT3A, and DNMT3B)


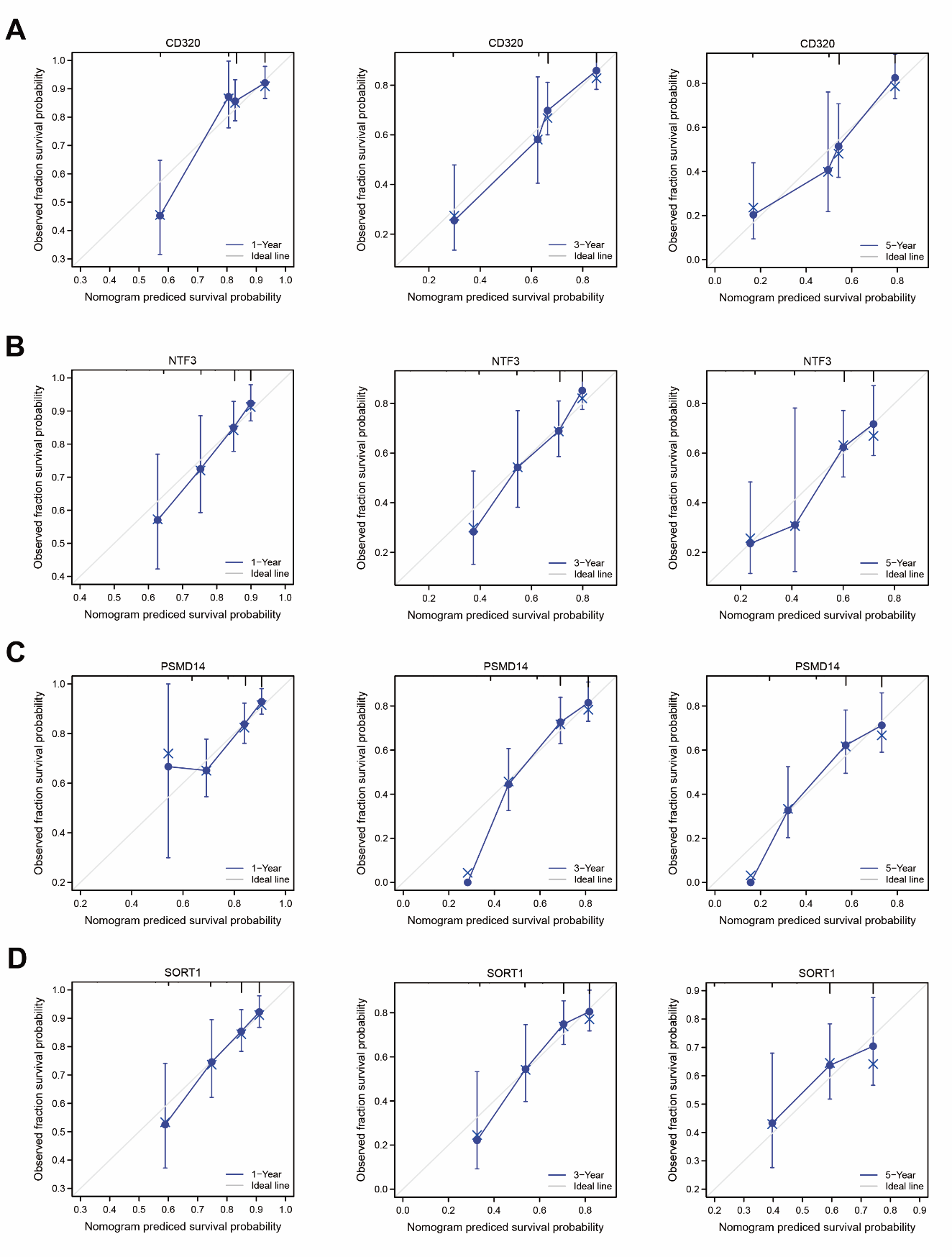


**Figure S7 (A-D)** The calibration analysis of the 4 prognostic nomogram models (CD320, PSMD14, SORT1, and NTF3)


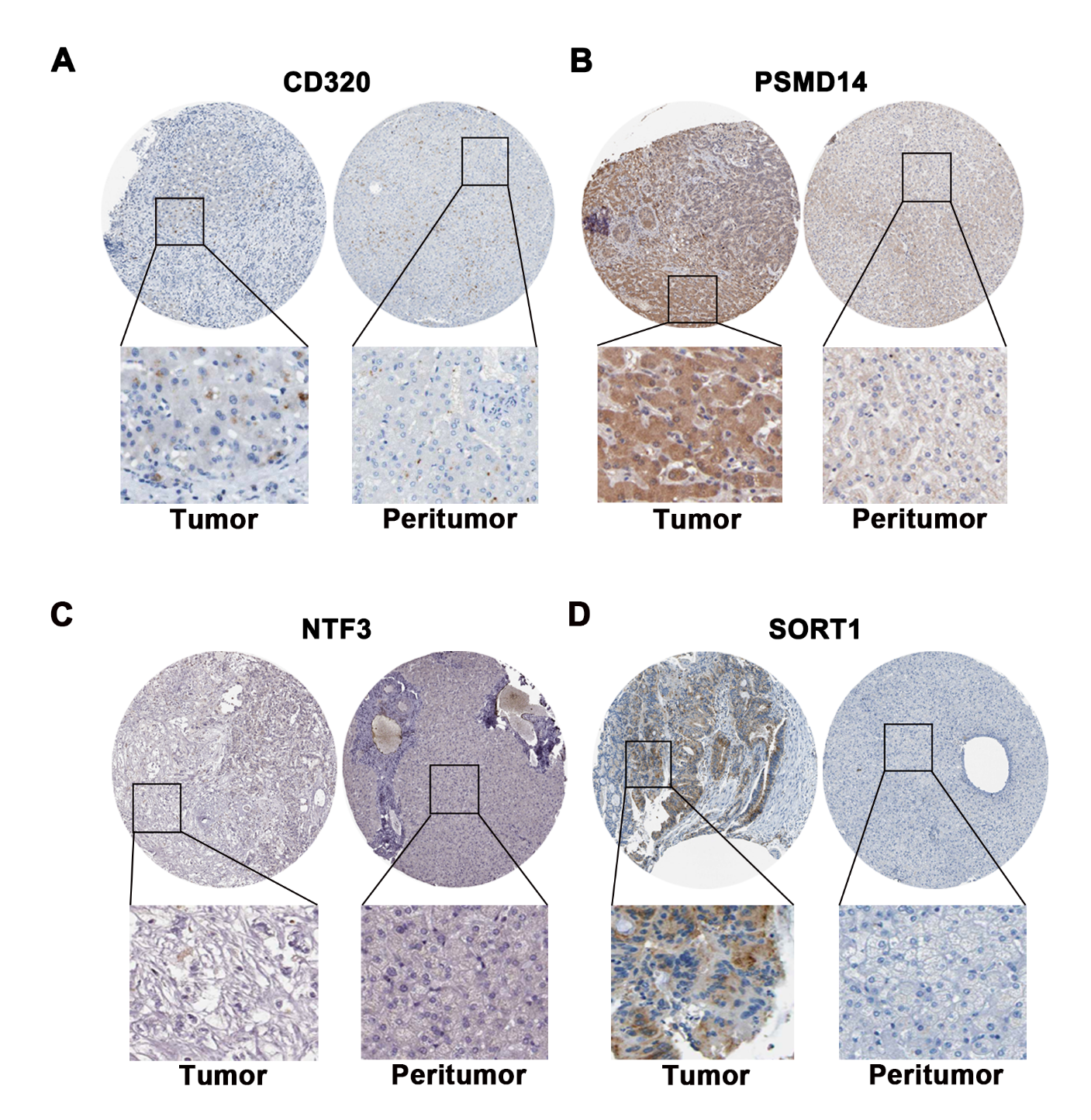


**Figure S8** **(A-D)** Immunohistochemistry validation of gene protein expression (CD320, PSMD14, NTF3, and SORT1) from the Human Protein Atlas database.


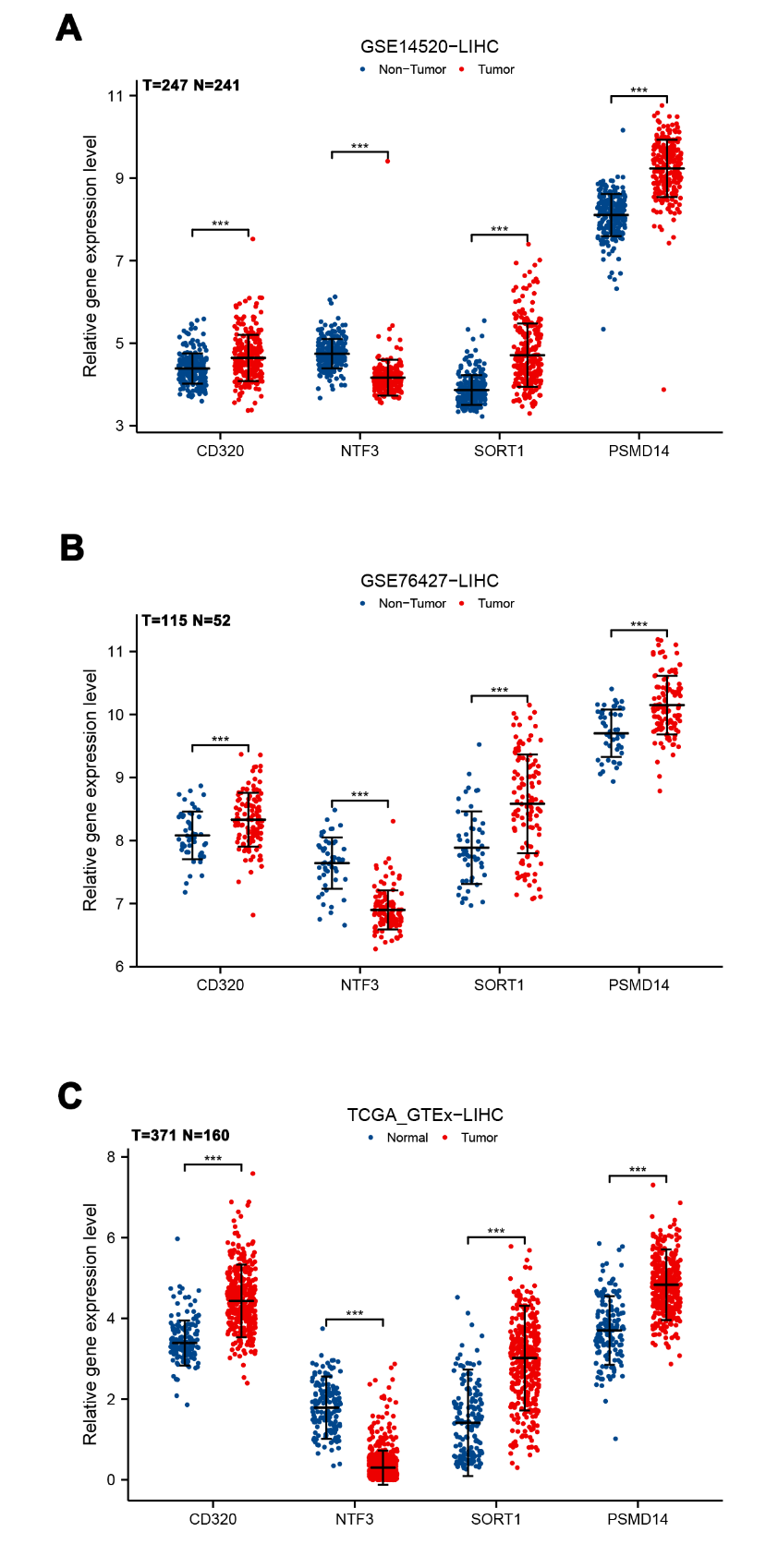


**Figure S9** Investigate the 4 hub genes (CD320, PSMD14, SORT1, and NTF3) expression level in the GSE14520 (T=247; N=241) **(A)**, GSE76427 (T=115; N=52) **(B)**, and TCGA (T=371; N=160) **(C)** expression matrix. (*P<0.05, **P<0.01, ***P<0.001, ****P<0.0001, n.s. not statistically significant)


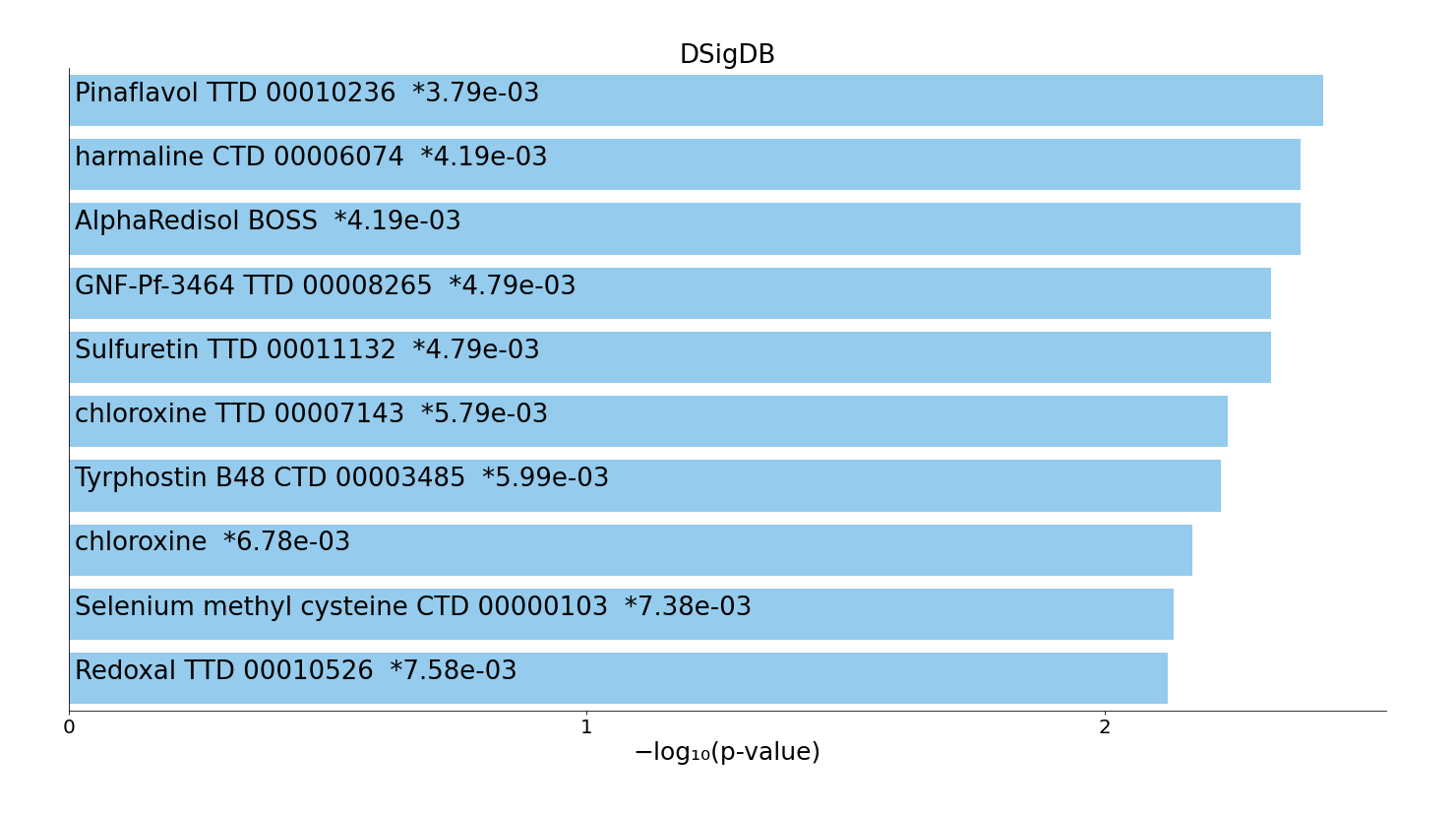


**Figure S10** Prediction of DEIRGs-related drugs.

## Additional Table

**Table S1: Prediction of DEIRGs-related top 10 drugs**

| Term | Adjusted P- value | Odds Ratio | Combined Score | Genes |
| --- | --- | --- | --- | --- |
| Pinaflavol TTD 00010236  harmaline CTD 00006074  AlphaRedisol BOSS  GNF-Pf-3464 TTD 00008265  Sulfuretin TTD 00011132  chloroxine TTD 00007143  Tyrphostin B48 CTD 00003485  chloroxine  Selenium methyl cysteine CTD 00000103  Redoxal TTD 00010526 | \| 0.003794816 \| \| --- \| \| 0.004193643 \| \| 0.004193643 \| \| 0.00479166 \| \| 0.00479166 \| \| 0.005787756 \| \| 0.005986886 \| \| 0.006783106 \| \| 0.007379957 \| \| 0.007578847 \| | \| 369.962963 \| \| --- \| \| 332.9333333 \| \| 332.9333333 \| \| 289.4637681 \| \| 289.4637681 \| \| 237.7142857 \| \| 229.5057471 \| \| 201.6464646 \| \| 184.8148148 \| \| 179.8108108 \| | \| 2062.217701 \| \| --- \| \| 1822.538786 \| \| 1822.538786 \| \| 1545.990778 \| \| 1545.990778 \| \| 1224.706508 \| \| 1174.652611 \| \| 1006.885371 \| \| 907.2536216 \| \| 877.9072583 \| | \| PSMD14 \| \| --- \| \| PSMD14 \| \| CD320 \| \| PSMD14 \| \| PSMD14 \| \| PSMD14 \| \| PSMD14 \| \| PSMD14 \| \| SORT1 \| \| PSMD14 \| |
